# *First do no harm* overlooked: Analysis of COVID-19 clinical guidance for maternal and newborn care from 101 countries shows breastfeeding widely undermined

**DOI:** 10.3389/fnut.2022.1049610

**Published:** 2023-01-17

**Authors:** Karleen Gribble, Jennifer Cashin, Kathleen Marinelli, Duong Hoang Vu, Roger Mathisen

**Affiliations:** ^1^School of Nursing and Midwifery, Western Sydney University, Parramatta, NSW, Australia; ^2^Alive & Thrive Southeast Asia, FHI 360, Washington, DC, United States; ^3^Department of Pediatrics, University of Connecticut School of Medicine, Hartford, CT, United States; ^4^Alive & Thrive Southeast Asia, FHI 360, Hanoi, Vietnam

**Keywords:** COVID-19, breastfeeding, policy, psychosocial support systems, rooming-in care

## Abstract

**Background:**

In March 2020, the World Health Organization (WHO) published clinical guidance for the care of newborns of mothers with COVID-19. Weighing the available evidence on SARS-CoV-2 infection against the well-established harms of maternal-infant separation, the WHO recommended maternal-infant proximity and breastfeeding even in the presence of maternal infection. Since then, the WHO’s approach has been validated by further research. However, early in the pandemic there was poor global alignment with the WHO recommendations.

**Methods:**

We assessed guidance documents collected in November and December 2020 from 101 countries and two regional agencies on the care of newborns of mothers with COVID-19 for alignment with the WHO recommendations. Recommendations considered were: (1) skin-to-skin contact; (2) early initiation of breastfeeding; (3) rooming-in; (4) direct breastfeeding; (5) provision of expressed breastmilk; (6) provision of donor human milk; (7) wet nursing; (8) provision of breastmilk substitutes; (9) relactation; (10) psychological support for separated mothers; and (11) psychological support for separated infants.

**Results:**

In less than one-quarter of country guidance were the three key breastfeeding facilitation practices of skin-to-skin contact, rooming-in, and direct breastfeeding recommended. Donor human milk was recommended in under one-quarter of guidance. Psychological support for mothers separated from their infants was recommended in 38%. Few countries recommended relactation, wet nursing, or psychological support for infants separated from mothers. In three-quarters of country guidance, expressed breastmilk for infants unable to directly breastfeed was recommended. The WHO and the United Kingdom’s Royal College of Obstetricians and Gynecologists were each cited by half of country guidance documents with the United States Centers for Disease Control and Prevention directly or indirectly cited by 40%.

**Conclusion:**

Despite the WHO recommendations, many COVID-19 maternal and newborn care guidelines failed to recommend skin-to-skin contact, rooming-in, and breastfeeding as the standard of care. Irregular guidance updates and the discordant, but influential, guidance from the United States Centers for Disease Control may have been contributory. It appeared that once recommendations were made for separation or against breastfeeding they were difficult to reverse. In the absence of quality evidence on necessity, recommendations against breastfeeding should not be made in disease epidemics.

## 1. Introduction

On 13 March 2020, the World Health Organization (WHO) published detailed clinical guidance on caring for infants of mothers with COVID-19 (1). In this guidance, the WHO stated that newborns should be placed skin-to-skin with their mothers after birth, initiate breastfeeding within an hour of birth, remain proximate to their mothers during the day and night, and exclusively breastfeed (1). When mothers were too ill to breastfeed, they should be supported to express milk and they and their infants should be provided with psychological support to mitigate the adverse effects of any separation that occurred (1). Additionally, mothers were to apply infection prevention and control (IPC) practices including wearing a mask, washing their hands, and cleaning surfaces they had been in contact with (1). Although the WHO has since updated their clinical guidance (2–4), these recommendations have remained unchanged.

The WHO followed a precautionary approach, weighing the limited knowledge on COVID-19 against the well-established harms of maternal-infant separation and determined that close mother-infant contact and breastfeeding should continue (5). They had learnt from the experience of the Human Immunodeficiency Virus (HIV) pandemic, in which it was demonstrated that seeking to prevent infection at all costs could result in more infant deaths than balancing all risks (6). The WHO’s COVID-19 recommendations for mothers and newborns therefore aligned with the standards of care of the WHO Early Essential Newborn Care Practices and the Baby-Friendly Hospital Initiative (7, 8). The WHO guidance reflected a recognition that not following these standards of care impedes breastfeeding and maternal attachment, leading to increased infant morbidity, mortality, and child maltreatment (9). In the longer term, reduced breastfeeding also increases maternal mortality from reproductive cancers and type II diabetes and has significant economic costs to societies related to health care costs, excess mortality, and cognitive losses from poorer child development (10).

Nonetheless, in an analysis we conducted of COVID-19 maternal and newborn care guidance from 33 countries collected 21 March 2020 to 30 April 2020 misalignment with the WHO recommendations was widespread (11). Most countries did not recommend keeping mothers with COVID-19 and their infants in close proximity or the practice of direct breastfeeding. It was uncommon to recommend psychological support for mothers separated from their infants and rare to recommend psychological support for infants separated from their mothers (11). The influence of recommendations from health agencies other than the WHO was evident in national guidelines and, where this differed from the WHO, was a source of confusion. In particular, the guidance of the United States Centers for Disease Control and Prevention (USCDC), which recommended maternal-infant separation, was commonly cited and was implicated in this confusion (11).

The negative effect of separating mothers and infants because of COVID-19 has been quantified. Bartick et al. (12) found that when infants of mothers with COVID-19 did not experience skin-to-skin contact they were 2.6 times more likely not to be exclusively breastfed up to 3 months of age than when they experienced skin-to-skin contact. They also found that when infants were kept in a separate room from their mothers, they were 3.8 times more likely to not be exclusively breastfed up to 3 months of age than infants who roomed in with their mothers. When mothers and infants were separated, 58% of mothers reported feeling very distressed and 29% of mothers who sought to breastfeed after reunification were unable to do so (12).

Rollins et al. (13) used the Lives Saved Tool to estimate the impact of policies separating mothers with COVID-19 and their infants on infant mortality in low- and middle-income countries. Using upper estimates of SARS-CoV-2 infection, transmission, and mortality, they calculated that maintaining maternal-infant proximity and breastfeeding when mothers had COVID-19 might result in a total of 2,800 infant deaths. In comparison, they estimated that infant mortality when mothers ceased breastfeeding temporarily or permanently due to policies of separation would result in 189,000–273,000 infant deaths (13).

Since the WHO first made recommendations on breastfeeding and newborn care in the context of COVID-19, there has been a substantial volume of research published. It has been confirmed that COVID-19 is rarely serious in infants (14), vertical transmission of SARS-CoV-2 during pregnancy or birth appears unlikely (15), and viable SARS-CoV-2 is not present in breastmilk (16). Further, it is unusual for infants to become infected with SARS-CoV-2 in the days after birth (17) and skin-to-skin contact, breastfeeding, and rooming-in do not increase the rate of COVID-19 symptoms in infants (12). Furthermore, mother-infant separation does not prevent infants from being infected with SARS-CoV-2 (16) as it exposes infants to the risk of nosocomial and other transmission routes (14). However, maintaining breastfeeding is likely to help protect infants against COVID-19 as breastmilk of mothers who have been infected with or vaccinated against COVID-19 contains antibodies against SARS-CoV-2 (18, 19). Further, SARS-CoV-2 is neutralized when added to the breastmilk of women with COVID-19 (20). And finally, the breastmilk of mothers infected with SARS-CoV-2 primes the infant’s own immune system to protect them against infection as shown by the presence of SARS-CoV-2 spike-specific secretory immunoglobulin A (IgA) and secretory immunoglobulin G (IgG) in infant saliva (21). Thus, the cautious approach of the WHO in recommending maternal and infant proximity and breastfeeding when mothers have COVID-19 has been validated. As knowledge about COVID-19 increased, it would be expected that country guidance would concomitantly improve in alignment with that of the WHO. This study aimed to assess global alignment with WHO recommendations and to assess how alignment had changed over time for the countries included in our previous analysis.

## 2. Materials and methods

### 2.1. Design

A critical integrative literature review of international COVID-19 guidance was undertaken. This design was chosen because it *“summarizes past empirical or theoretical literature to provide a more comprehensive understanding of a particular phenomenon or healthcare problem”* (22), p. 546]. In this case, the problem was a lack of knowledge on the degree to which COVID-19 country guidance for breastfeeding and newborn care aligned with WHO recommendations and how alignment had changed over time.

### 2.2. Sample

One hundred and eighty-three COVID-19 guidance documents from 108 countries on six continents containing content on pregnancy, intrapartum, and postpartum care in the context of COVID-19 were reviewed for inclusion in the study. A hierarchy of inclusion for guidance was followed with national government guidance prioritized, followed by state/provincial government guidance, and then professional medical association guidance. Where there was more than one government guidance document identified, all current guidance were included for analysis. Where guidance from multiple professional organizations was identified, guidance from the obstetrics and gynecologists’ association was prioritized over pediatricians’ association guidance. Where a country’s government guidance only addressed breastfeeding or was targeted at mothers rather than clinicians, professional organizational guidance was included if available. If there was significant uncertainty that guidance was current, it was excluded. If newer versions of guidance were published after the collection period, they were not included in the analysis.

### 2.3. Data Collection

International guidance documents on pregnancy, intrapartum, and postpartum care in the context of COVID-19 were collected between 15 November 2020 and 31 December 2020. Guidance documents were specifically sought from all countries that are members of the World Health Assembly (WHA) but guidance from other countries or organizations encountered in searches were also included. In the first instance, the websites of the Ministries of Health in each country were searched for guidance. Guidance published on Ministry of Health websites was assumed to be current unless otherwise stated. Where guidance (or direction to guidance published elsewhere) could not be located on Ministry of Health websites, country contacts were asked to assist in identifying guidance. Where country contacts could not locate guidance, the Ministry of Health, Minister for Health, and/or United Nations Children’s Fund (UNICEF) country offices were contacted and asked to assist in locating guidance. Where guidance documents stated that practices in specific and named external documents should be followed (for example the national infant feeding guidance or guidance from a professional association) the recommendations of those documents were included in the analysis. After evaluation against the inclusion and exclusion criteria, guidance documents from 101 countries and two regional agencies were included in the analysis. Where necessary, translation of guidance was undertaken by *Alive & Thrive* staff or by other individuals working in maternal and infant health known to the authors.

### 2.4. Data Analysis

Each guidance document was initially assessed and coded for alignment with the WHO *Clinical Management of COVID-19: Interim Guidance*, 27 May 2020 (2) by two authors. Any discrepancies were then discussed by all authors as a group and coding decided by consensus. Recommendations were coded regarding: (1) Skin-to-skin contact (S2S); (2) Early initiation of breastfeeding (EIBF); (3); Rooming-in (RI); (4) Direct breastfeeding (BF); (5) Provision of expressed breastmilk (EBM); (6) Provision of donor human milk (DHM); (7) Wet nursing (WN); (8) Provision of breastmilk substitutes (BMS); (9) Relactation (R); (10) Psychosocial support for separated mothers (PS-M); and (11) Psychosocial support for separated infants (PS-I). The WHO alignment scores were calculated for each guidance document. For each recommended practice, a score of 1 indicated alignment and a score of 0 indicated divergence from the WHO recommendation. The highest possible score was 11 and the lowest 0.

The practices of skin-to-skin contact, early initiation of breastfeeding, and direct breastfeeding were coded as recommended when guidance was unambiguously supportive of the practice. Where skin-to-skin contact, early initiation of breastfeeding, and direct breastfeeding were supported only on maternal/family request, after a discussion of risk, or with the decision to be made by health providers, they were coded as not recommended and the circumstances under which the practice was supported were noted. Where it was recommended that infants be isolated from their mothers, skin-to-skin contact, early initiation of breastfeeding, and direct breastfeeding were assumed impossible and coded as not recommended unless otherwise stated. For coding rooming-in, the Baby-Friendly Hospital Initiative definition requiring that infants remain proximate to their mothers, share a bed, be placed in a side-car attached to her bed, or in a crib directly beside her bed, was used (23). Recommendations allowing mothers to room-share with infants at a distance or infants to be kept in an incubator or behind a screen were coded as not recommending rooming-in with a notation made on the recommendation for physical distancing. Similarly, when rooming-in was provided only on maternal/family request or after a discussion of risk or with the decision to be made by health providers, rooming-in was coded as not recommended with an appropriate notation. When rooming-in was provided only if there were no facilities to permit maternal-infant separation, rooming-in was coded as not recommended. Requirements for documentation or provision of maternal written consent for skin-to-skin contact, rooming-in, room-sharing, or breastfeeding were noted.

Alternative feeding methods were coded based on whether recommendations for use prioritized breastmilk options. Recommendations regarding the use of expressed breastmilk were coded as in alignment with the WHO recommendations where guidance was unambiguously supportive if mothers were not directly breastfeeding. If use of expressed breastmilk was conditionally supported, it was coded as not in alignment with the WHO recommendations with a notation on reasons. Recommendations regarding donor human milk were coded as in alignment with the WHO recommendations where guidance supported use when maternal breastfeeding or expressed breastmilk were unavailable. If donor human milk was specified as only available for premature infants this was coded as contrary to the WHO recommendations. Recommendations regarding use of breastmilk substitutes were coded as in alignment with the WHO recommendations when they specified that use was supported if maternal expressed breastmilk was unavailable. Conversely if breastmilk substitutes were prioritized over, or equal to breastmilk, this was coded as contrary to the WHO recommendations.

Recommendations for psychological support for mothers were coded in alignment with the WHO recommendations regardless of whether it was connected to separation from infants. Recommendations that infants separated from their mothers should be provided with a specific alternative caregiver were included as recommending psychological support for infants.

When there was no information about whether a practice was recommended or not, it was coded as “no recommendation made.” When a conflict in recommended practices was identified within the same document, the recommendation that most differed from the WHO guidance was coded and included for analysis. When guidance had different recommendations based on maternal symptoms, the recommendation for mothers who had the most severe symptoms but were still physically capable of infant care was coded. When conflicts between guidance from the same country or idiosyncratic recommendations were identified through the guidance collection process, they were noted. Recommendations for the washing of breasts were noted. References to guidance documents from other countries within the country guidance were recorded as were publication dates of guidance.

Recommendations of 32 countries’ guidance that we previously assessed (11) were compared to the guidance from the same countries in the current data set. In this comparison, Malawi was excluded because updated guidance or confirmation of currency could not be obtained. In addition, the score for the Canadian guidance was adjusted to take into account different treatment of external guidance in the coding of the current study. In the earlier research, the Canadian national infant feeding recommendations were included in the coding as the COVID-19 guidance had recommended that “standard practice” be followed. However, in the current study, external guidance was only coded where a specific document was named and so the national infant feeding recommendations were not included.

## 3. Results

**FIGURE 1:**
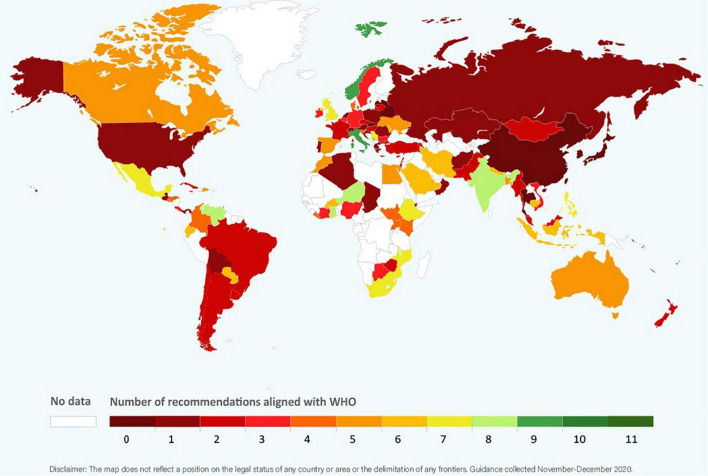
Global alignment with WHO recommendations (score 0 to 11; data from 101 countries).

Clinical guidance from 77 government agencies, 24 professional medical associations, and two regional agencies were included in our analysis (Supplementary Table 1). Nineteen (18.5%) were from Africa, 31 (30.1%) from Asia, three (2.9%) from the Caribbean, 18 (17.5%) from Central, North, and South America, 29 (28.2%) from Europe and three (2.9%) from Oceania. Ninety-nine countries from which guidance was obtained were members of the WHA and two (Kosovo, Taiwan) were not members. The regional guidance documents were from the Pan American Health Organization (PAHO) and the Pacific Joint Incident Management Team (PJIMT). Among the guidance reviewed, only that from PAHO was fully aligned with the WHO. The WHO alignment scores for countries ranged from 0 (Belarus, China, Latvia, Japan, Singapore, Slovakia, South Korea, Taiwan, and Thailand) to 9 (Italy and Norway) (Figure 1). Detailed information on alignment of each guidance is listed in Supplementary Table 2.

### 3.1. Skin-to-skin contact, early initiation of breastfeeding, direct breastfeeding and maternal proximity

**FIGURE 2:**
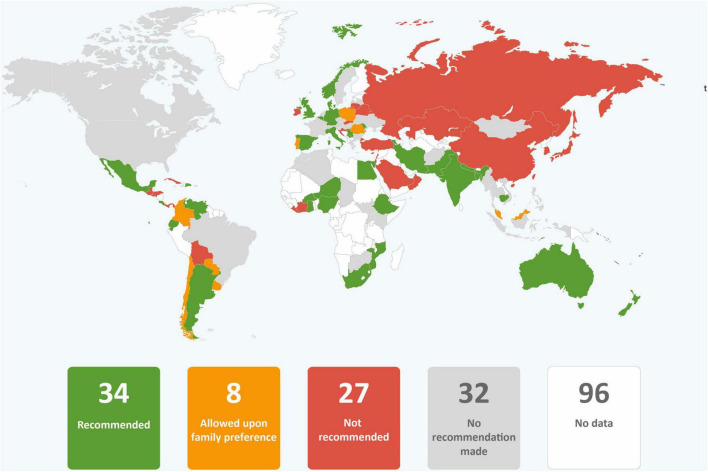
Global distribution of recommendations on skin-to-skin contact for infants of mothers with COVID-19 (data from 101 countries).

**FIGURE 3:**
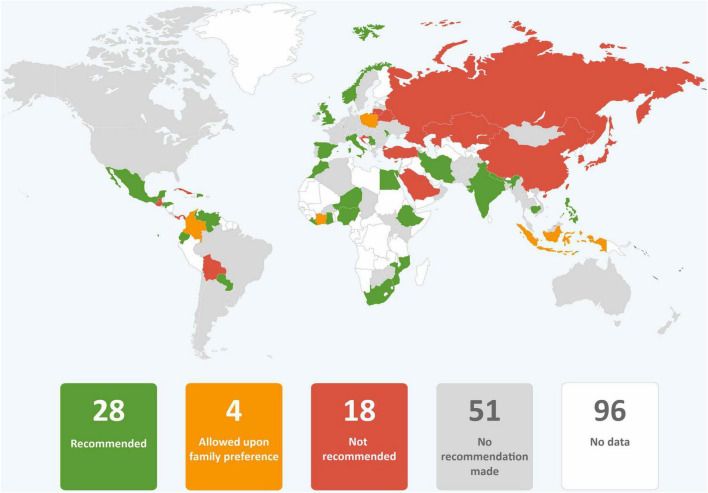
Global distribution of recommendations on early initiation of breastfeeding for infants of mothers with COVID-19 (data from 101 countries).

**FIGURE 4:**
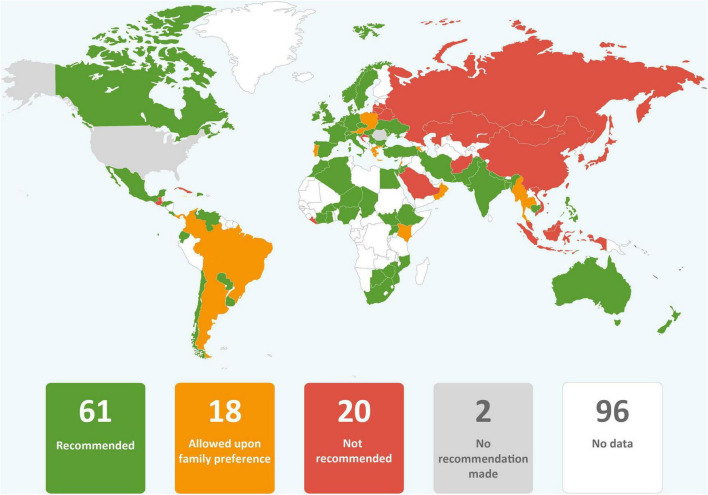
Global distribution of recommendations on direct breastfeeding for infants of mothers with COVID-19 (data from 101 countries).

In just over one third of the guidance reviewed, skin-to-skin contact was recommended, and in just less than one third of guidance early initiation of breastfeeding for babies born to mothers with COVID-19 was recommended. It was common for guidance to provide no recommendations regarding these practices. Direct breastfeeding was more commonly supported than either skin-to-skin contact or early initiation of breastfeeding and was recommended in nearly two thirds of the guidance (Table 1 and Figures 2–4).

**TABLE 1:** Number and frequency of guidance recommending, not recommending, and providing no recommendation on skin-to-skin contact, early initiation of breastfeeding, direct breastfeeding, rooming-in, relactation, and provision of psychological support for mothers and infants (*N* = 103).

Practice	Recommended *n* (%)	Not recommended ^a^ *n* (%)	Absent/No recommendation *n* (%)
Skin-to-skin contact	36 (35.0)	36 (35.0)	31 (30.1)
Early initiation of breastfeeding	30 (29.1)	23 (22.3)	50 (48.5)
Direct breastfeeding	63 (61.2)	38 (36.9)	2 (1.9)
Rooming-in	35 (34.0)	58 (56.3)	10 (9.7)
Relactation	11 (10.7)	0 (0.0)	92 (89.3)
Psychological support for mothers	39 (37.9)	0 (0.0)	64 (62.1)
Psychological support for infants	9 (8.7)	0 (0.0)	94 (91.3)

^a^Includes all categories except for “recommended” and “no information” (Not recommended; Allowed with 2 meters distance or with family preference).

**FIGURE 5:**
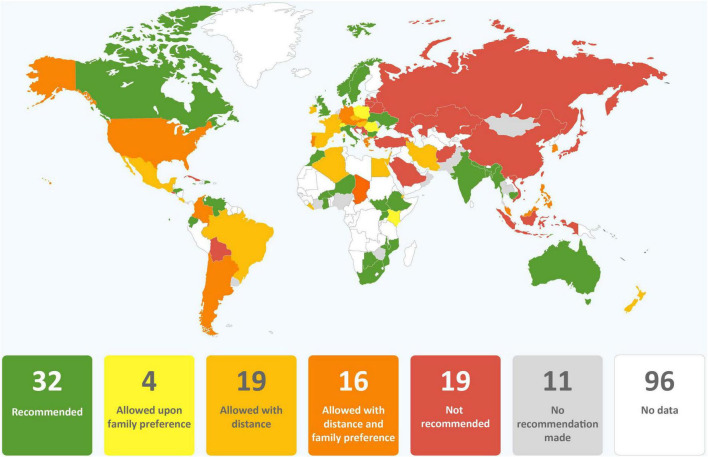
Global distribution of recommendations for rooming in for infants of mothers with COVID-19 (data from 101 countries).

Different degrees of maternal-infant proximity for women with COVID-19 were recommended in guidance documents ranging from rooming-in, to conditional rooming-in or room-sharing if the family requested (with risks discussed), room-sharing with the infant kept two meters distance from the mother, to complete isolation of the infant from their mother. Only one third of guidance unequivocally recommended rooming-in for mothers with COVID-19 and their infants. Nineteen (18.4%) guidance recommended isolation of infants from their mothers. Of the 39 (37.9%) sets of guidelines that permitted rooming-in or room-sharing only under specific circumstances, in 19 (18.4%) of these cases, maternal, family consent or health professional consent was required. In some of these guidance documents, isolation of infants from mothers was a clear expectation and rooming-in or room-sharing were provided only if mothers or parents refused. For example, the Bahrain National Taskforce COVID-19 National Protocols recommended that, *“Temporary separation between the mother and the newborn minimizes the risk of transmission and is advised. If parents refuse separation and willing to room in together, then precautions should be taken to minimize risk of viral transmission”* (24), p. 51]. Guidance on maternal proximity was absent in ten (9.7%) countries’ guidance (Table 1 and Figure 5).

In total, only 21 national guidance documents (20.4%) (Australia, Bangladesh, Burkina Faso, Cambodia, Denmark, Dominican Republic, Ecuador, Ethiopia, Ghana, India, Italy, Kosovo, Moldova, Mozambique, Nepal, Niger, Norway, South Africa, Timor-Leste, United Kingdom (UK), and Venezuela) and guidance from two regional agencies (PAHO and PJMIT) recommended the three core breastfeeding-enabling practices of skin-to-skin contact, direct breastfeeding, and rooming in.

### 3.2. Relactation

**FIGURE 6:**
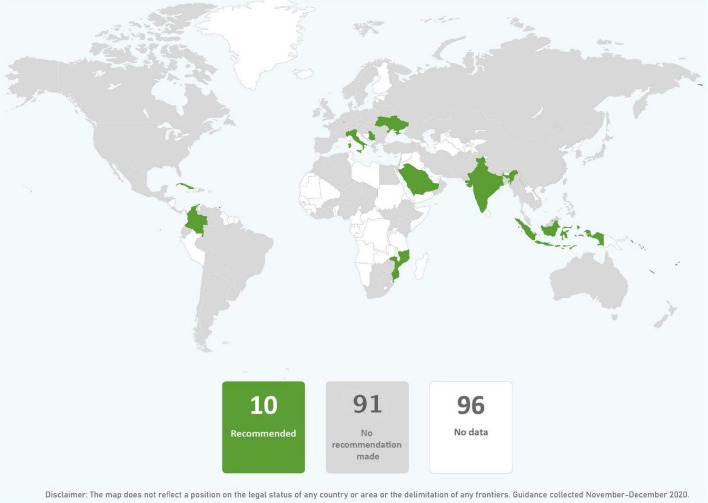
Global distribution of recommendations on relactation support for mothers with COVID-19 (data from 101 countries).

Only 11 guidance documents (10.7%) recommended that mothers be supported to relactate if separation or severe illness had resulted in lactation cessation (Table 1 and Figure 6).

### 3.3. Psychological support

**FIGURE 7:**
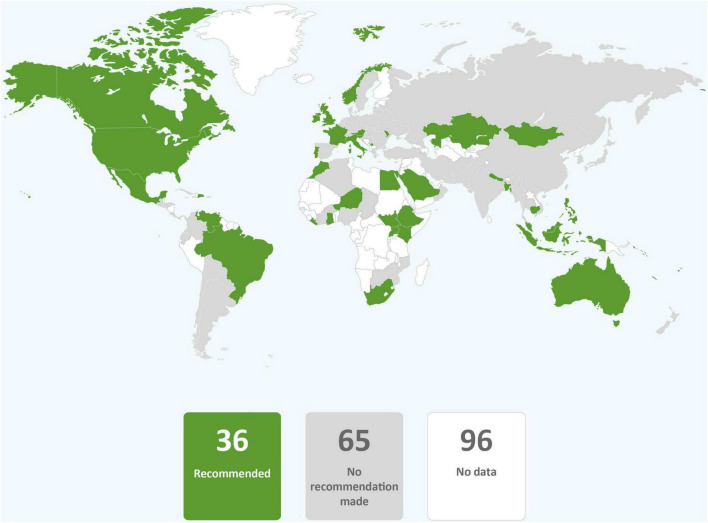
Global distribution of recommendations on psychological support for mothers with COVID-19 (data from 101 countries).

**FIGURE 8:**
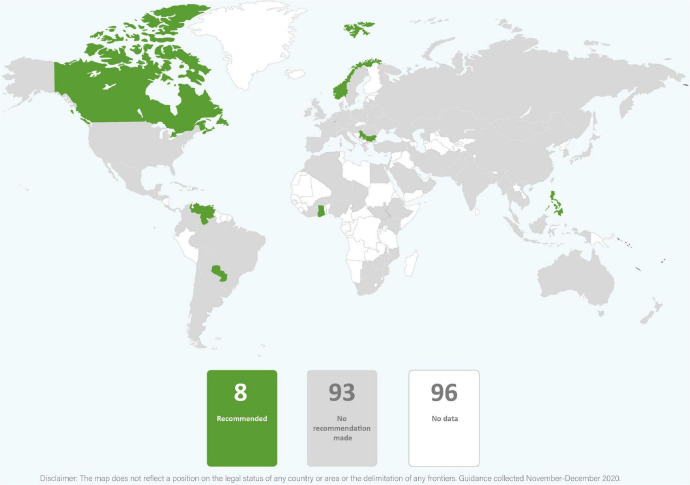
Global distribution of recommendations on psychological support for infants of mothers with COVID-19 (data from 101 countries).

While more than one third of guidance documents recommended the provision of psychological support for mothers, less than 10% provided a recommendation for psychological support for separated infants (Table 1 and Figures 7, 8). Among the guidance that recommended isolation of infants from their mothers with COVID-19, only three (2.9%) also recommended psychological support for separated mothers (Indonesia, Kazakhstan, and Saudi Arabia) and only one (1.0%) recommended psychological support for separated infants (Serbia). Guidance varied in how much information they included on how to provide support for separated mothers and infants. The Philippines Ministry of Health was among those that included more information stating that mothers should be provided with, *“Psychosocial/mental health support, lactation and maternal nutrition counseling, and practical infant feeding support, especially for those who may need to be separated from the newborn”* (25), p5]. The Paraguay Ministry of Public Health and Social Welfare provided detail on support for infants with a separate section in their guidance entitled, *“Choosing a companion for the newborn”* noting that this person should be someone the mother *“trusts to provide emotional support and help in the care of the newborn”* and stating that they should be trained in infant care including, *“infant hunger cues and administration of expressed breast milk by cup, spoon or finger, diapering, bathing, dressing, sleeping”* (26), p. 2].

### 3.4. Alternative feeding methods

**FIGURE 9:**
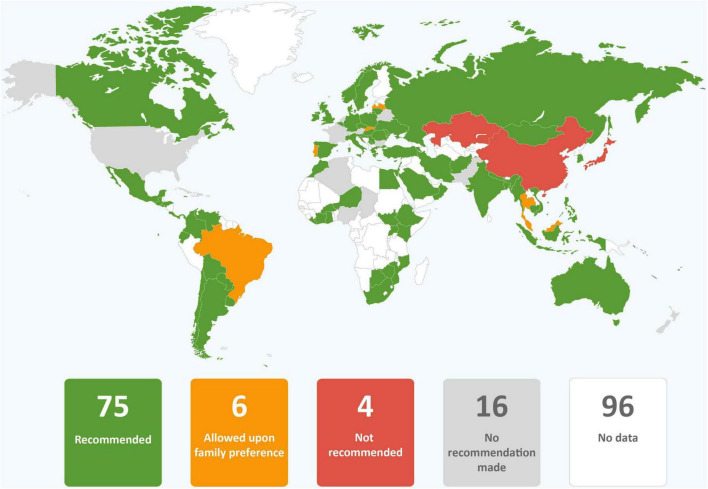
Global distribution of recommendations on the provision of expressed breastmilk to infants of mothers with COVID-19 unable to directly breastfeed (data from 101 countries).

**FIGURE 10:**
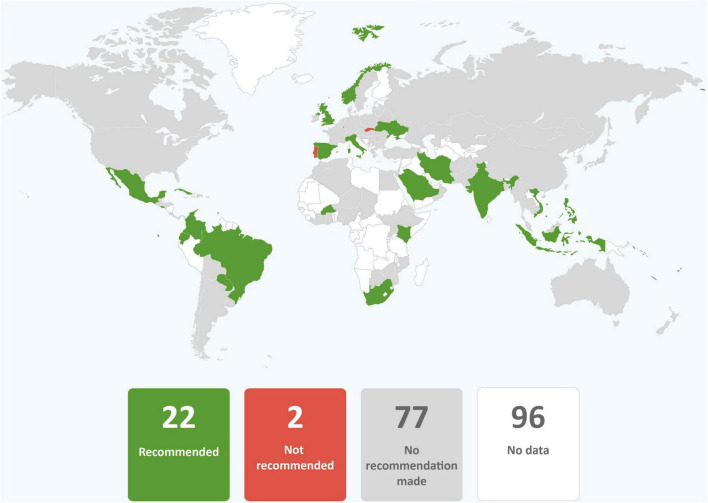
Global distribution of recommendations on providing donor human milk to infants of mothers with COVID-19 unable to provide their own breastmilk (data from 101 countries).

**FIGURE 11:**
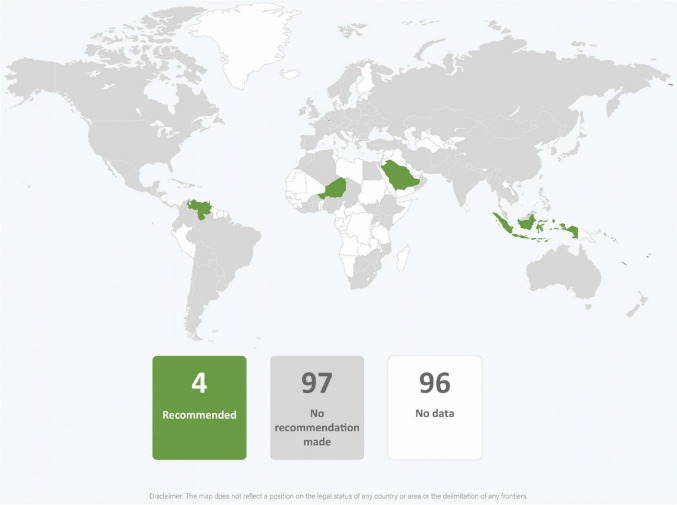
Global distribution of recommendations on wet nursing infants of mothers with COVID-19 (data from 101 countries).

**FIGURE 12:**
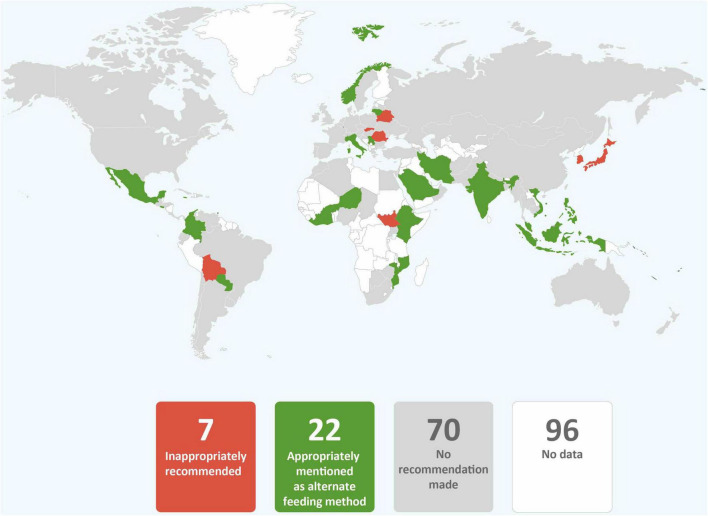
Global distribution of recommendations to provide breastmilk substitutes to infants of mothers with COVID-19 only where breastfeeding or expressed breastmilk is not available (data from 101 countries).

In most guidance, providing expressed breastmilk to infants was recommended when mothers and infants were separated or direct breastfeeding was not recommended because of maternal COVID-19 status (Table 2 and Figure 9). However, guidance from eleven countries (9.7%) recommended against or provided only conditional support for feeding expressed breastmilk from mothers with confirmed COVID-19 (Brazil, China, Japan, Kazakhstan, Latvia, Malaysia, Portugal, Singapore, Slovakia, South Korea, and Thailand). Donor human milk was commonly absent as an alternative feeding method and recommended by just under a quarter of country guidance (Table 2 and Figure 10). None of the countries that recommended against expressed breastmilk feeding when mothers had COVID-19, recommended donor human milk be provided. It was rare for wet nursing to be recommended as an alternative feeding method and this was the recommendation of the WHO least adopted by countries (Table 2 and Figure 11). Indeed, only four countries and one regional agency (PAHO) recommended wet nursing for infants unable to access their own mothers’ breastmilk. Appropriate recommendations for feeding infants with breastmilk substitutes when breastfeeding or provision of expressed breastmilk was not possible, were included in 25 (24.3%) guidance documents. Seven (6.8%) country guidance documents specifically recommended feeding breastmilk substitutes to infants born to mothers with COVID-19 in preference to breastfeeding or feeding expressed breastmilk (Figure 12). Although breastmilk substitute feeding formed a part of the recommendations of many countries, few countries provided detailed guidance on management of safe infant formula feeding (data not shown) and none discussed the need to ensure that families had the resources to access, purchase, and properly prepare infant formula after hospital discharge.

**TABLE 2:** Number and frequency of guidance documents recommending, not recommending, and providing no recommendation on alternative feeding methods of expressed breastmilk, donor human milk, and wet nursing (*N* = 103).

Practice	Recommended *n* (%)	Not recommended *n* (%)	Absent/No recommendation *n* (%)
Expressed breastmilk	76 (73.8)	11 (9.7)^a^	16 (15.5)
Donor human milk	23 (22.3)	2 (1.9)	78 (75.7)
Wet nursing	5 (4.9)^b^	0 (0.0)	98 (95.1)

^a^Includes “not recommended” and “allowed upon family request.”

^b^Includes four countries and one regional agency (PAHO).

### 3.5. Breast washing

Fifteen (14.6%) countries inappropriately recommended breast washing before breastfeeding while six (5.8%) countries recommended breast washing only if breasts had been coughed on (in line with the WHO recommendations). Guidance from the Philippines noted that breast washing should not be undertaken as long as IPC measures were followed. Statements regarding what to wash breasts with were rare but Iran was an exception with guidance from the Ministry of Health stating to *“avoid washing the breast with disinfectants, especially alcohol-based ones”* (27), p. 6]. Three (2.9%) countries recommended breast washing before expressing milk.

### 3.6. Guidance referenced

The most frequently referenced organizational guidance in guidance in our dataset (*N* = 103) were from the WHO [*n* = 51, (49.5%)], the Royal College of Obstetricians and Gynecologists and Royal College of Midwives (RCOG) (*n* = 50, 48.5%), the USCDC (*n* = 33, 32.0%), and the American College of Obstetricians and Gynecologists (ACOG) (*n* = 20, 19.4%). The ACOG guidance simply reiterated the USCDC and directed readers to the USCDC guidance and either one or both of these organizational guidelines were referenced by guidance from *n* = 40 (38.8%) countries. Thirty-eight (36.9%) countries’ government guidance documents did not reference the WHO, of these 12 (11.7%) referenced no external guidance. The confusion created by the conflicting recommendations of the WHO and RCOG (both of which recommended maternal proximity and breastfeeding) and the USCDC (which did not) was evident in some country guidance. For example, the guidance from the Royal Thailand College of Obstetrics and Gynecology noted that the WHO, the RCOG as well as guidance from Canada, Australia and New Zealand and “most European countries” recommended maternal proximity and breastfeeding. However, they also state that the USCDC recommended maternal-infant separation. This guidance document then provided no recommendation on breastfeeding but instead stated that mothers should be provided with information on the pros, cons, and risks of breastfeeding their infants before making a decision.

### 3.7. Conflicts, lack of clarity, and anomalies within guidance documents

Conflicts and confusion were present in some countries’ guidance documents. For example, guidance from Honduras recommended that no skin-to-skin contact be provided but also said that the WHO Early Newborn Care Practices (which includes skin-to-skin contact) should be followed. Guidance from Afghanistan recommended both for and against direct breastfeeding. The Saudi Arabia government published a series of guidance documents and did not withdraw older guidance when publishing new documents. This resulted in contradictory recommendations between guidance documents, for example, against and for separation and direct breastfeeding. The Kazakhstan Ministry of Health guidance was among several that was difficult to interpret because it provided different and unclear recommendations for different scenarios.

Many guidance documents abstained from mentioning key practices altogether (for example skin-to-skin contact), while others discussed practices but declined to provide a recommendation. For example, the USCDC discussed but did not provide recommendations on rooming-in or breastfeeding and was among eight countries whose guidelines provided no recommendations on specific practices but instead stated that mothers or families should be informed of the risks and benefits and make their own decision. The Malaysian Ministry of Health guidance was among several that recommended that individual hospitals should develop their own guidelines on specific practices.

Eight (7.8%) countries stated that mothers should be required to provide written consent for practices recommended by the WHO including skin-to-skin contact, maternal proximity, provision of expressed breastmilk, and breastfeeding. In some cases, a consent form which clearly implied that proximity and breastfeeding were risky was included. For example, the consent form for direct breastfeeding in the guidance of the Guatemalan Institute of Social Security stated, *“Having had the risks involved and alternatives for breastfeeding explained, I TAKE RESPONSIBILITY AND ASSUME THE RISKS THAT WERE EXPLAINED TO ME AND THE COMPLICATIONS THAT THIS MAY CAUSE IN MY BABY’S HEALTH, I SIGN SAID CONSENT”* (emphasis in original) (28), p. 32]. In only one country guidance was consent required for a practice that was not in line with WHO recommendations (Slovakia for separation). The issue of the rights of the mother-newborn dyad regarding proximity was raised only in the guidance from Kosovo and Cambodia both of which said, *“Every newborn has the right to access his or her mother or parent. No mother should be separated from her baby without her informed consent”* (29), p. 71, (30), p. 33].

A variety of questionable or impractical recommendations were made. Guidance from Brazil, Columbia, and Mexico recommended that mothers avoid talking during breastfeeding. Guidance from Costa Rica stated that mothers should put on a clean gown prior to breastfeeding. Guidance from Brazil stated that infants should be prevented from touching their mother’s face. Guidance from Côte d’Ivoire, Germany, Hungary, Paraguay, and South Africa advised that mothers with COVID-19 not kiss their babies. Guidance from South Korea recommended testing the breastmilk of women with COVID-19 for SARS-CoV-2 and to consider feeding expressed breastmilk only after confirmation of a negative result. Seemingly in response to poor practices and misinformation by others, guidance from Djibouti, France, Iran, the Philippines, and the UK noted that masks should not be put on infants.

### 3.8. Publication dates of guidance

Guidance from 95 (92.2%) countries were dated, with the first guidance published 9 February 2020 (China) and the latest published or updated in December 2020 (Iran and Malaysia). More than half of countries’ whose guidance had publication dates were either published or most recently updated between March and May 2020 (*n* = 48, 50.5%) (Supplementary Figure 1).

### 3.9. Changes in recommendations

In the 32 countries for which comparison between the recommendations of March-April 2020 and November-December 2020 could be made, 16 (50%) countries increased in alignment with the WHO recommendations; three countries (9.4%) decreased in alignment; and 13 (40.6%) remained the same (Supplementary Figure 2). In six countries (18.6%) (China, Jamaica, Myanmar, Nigeria, Singapore, and Vietnam), alignment remained the same because the guidance had not been updated at the time of data collection. Some country alignment scores increased markedly with Ethiopia improving by seven and India, Italy, and the Philippines improving by six. The proportion of individual recommendations in alignment with the WHO increased for all recommendations when compared to both the 32 countries for which direct comparison could be made and overall for the 103 country and regional guidance in the November-December 2020 data set (Table 3).

**TABLE 3:** Frequency of country and regional guidance in alignment with each of the eleven World Health Organization (WHO) COVID-19 recommendations for maternal and newborn care in guidance collected March-April 2020 and November-December 2020.

	Percentage of guidance in alignment with the WHO recommendation										
	S2S ^a^ *n* (%)	EIBF *n* (%)	RI *n* (%)	BF *n* (%)	EBM *n* (%)	DHM *n* (%)	WN *n* (%)	BMS *n* (%)	PS-M *n* (%)	PS-I *n* (%)	R *n* (%)
March–April 2020 collection (*n* = 32)	7 (21.9)	5 (15.6)	11 (34.4)	15 (46.9)	23 (71.9)	3 (9.4)	0 (0.0)	6 (18.8)	4 (12.5)	1 (3.1)	2 (6.3)
November–December 2020 collection (*n* = 32 ^b^)	13 (40.6)	10 (31.3)	12 (37.5)	18 (56.3)	22 (68.8)	13 (40.6)	2 (6.3)	14 (43.8)	19 (59.4)	3 (9.4)	4 (12.5)
November–December 2020 collection (*n* = 103 ^c^)	36 (35.0)	30 (29.1)	35 (34.0)	63 (61.2)	76 (73.8)	23 (22.3)	5 (4.9)	25 (24.3)	39 (37.9)	9 (8.7)	11 (10.7)

^a^S2S, skin-to-skin contact; EIBF, early initiation of breastfeeding; RI, rooming in; BF, direct breastfeeding; EBM, expressed breastmilk; DHM, donor human milk; WN, wet nursing; BMS, breastmilk substitutes; PS-M, psychological support for mothers; PS-I, Psychological support for infants; R, relactation; ^b^same countries as in March-April 2020 collection; ^c^full data set.

## 4. Discussion

In this analysis of COVID-19 maternal and newborn guidance documents, we revealed that alignment of country recommendations with the recommendations of the WHO regarding breastfeeding and related practices improved during 2020. Alignment particularly improved for skin-to-skin contact, early initiation of breastfeeding, direct breastfeeding, donor human milk when mother’s milk is unavailable, and psychological support for mothers separated from their infants.

However, it is concerning that approximately 9 months after the WHO first published their clinical guidance and after a substantial volume of research on COVID-19 had been published, alignment with the WHO recommendations remained at an overall low level. Of the 103 guidance documents collected from November through December 2020, nearly two thirds recommended direct breastfeeding, but only one third recommended skin-to-skin contact and one third recommended rooming-in. Alarmingly, less than a quarter of this guidance unequivocally recommended the three key practices that support breastfeeding, namely: skin-to-skin contact, direct breastfeeding, and rooming-in (9). Thus, country guidance placed many mothers in a situation where they were advised to breastfeed but simultaneously denied support for practices that facilitate and enable breastfeeding. To recommend breastfeeding while putting in place structural barriers to breastfeeding is unfair to women (31). It also results in reduced exclusivity and duration of breastfeeding (9). The fact that 20% of guidance documents required that infants be isolated from mothers with COVID-19 placed women at a very high risk of being unable to breastfeed in many countries (12). While in the remaining nearly 40% of guidance, rooming-in or room-sharing was permitted when mothers or families wanted it, it was evident in some guidance documents that this was not simply a case of mothers stating their preferences. Rather, mothers would need to strongly advocate to be allowed to room-in or room-share with their infants. Requirements for mothers to provide written consent is reflective of the representation of rooming-in (as well as skin-to-skin contact and breastfeeding) as risky practices rather than a part of the standard of care. In some contexts, these requirements may have pre-dated COVID-19, evidencing already weak support for breastfeeding that continued or may have deteriorated during the pandemic. As an example, prior to the pandemic, the Japanese Society of Perinatal and Neonatal Medicine stated that pregnant women be provided with information on the risks and benefits of skin-to-skin contact with the practice to be implemented recorded in medical records (32).

Health workers are obligated to provide care in a manner that does no harm. Improved policies and redesigned processes of care are among several low-cost strategies that protect patient safety and prevent adverse health outcomes (33, 34). In our analysis, the absence of recommendations for evidence-based practices, including where statements were made that mothers should be provided with information on the risks and benefits and make their own decision, represents an abandonment of responsibility by health authorities toward mothers and health providers. In practice, it means that mothers must rely on the knowledge of individual health professionals. The HIV pandemic demonstrated that lack of clear infant feeding recommendations compromises the ability of health professionals to support mothers in decision making (35) and places disproportionate emphasis on the opinions of individual health professionals (36) to the detriment of mothers and infants. It is concerning that so many health authorities abrogated their responsibility in this way.

Feeding infants their mothers’ expressed breastmilk if they were unable to directly breastfeed was the most common recommendation that aligned with the WHO guidance remaining the same between March-April 2020 and November-December 2020. However, one quarter of guidance documents did not recommend feeding infants expressed breastmilk and either had no recommendation, recommended against, or recommended only feeding expressed milk if it was a maternal/family preference or after testing. Given that there is no evidence of replicable SARS-CoV-2 in breastmilk, or that the virus could be transmitted *via* breastmilk, the absence of an unconditional universal recommendation to feed expressed breastmilk to infants who are unable to directly breastfeed, is contrary to public health principles.

The number of recommendations in favor of using donor human milk increased between March-April 2020 and November-December 2020. This may have partly been a result of advocacy by organizations like the Global Alliance of Milk Banks and Associations (formed early in the pandemic) and others (37, 38). The proportion of guidance recommendations in favor of relactation also increased, doubling from the first to the second collection of guidance documents. However, the overall proportion of guidance documents recommending relactation remained low with only one in nine guidance documents including the practice. The potential impact of relactation and motivation to relactate during the pandemic has been described. Rollins et al. (13) calculated that where mothers with COVID-19 in low- and middle-income countries were separated from their infants and ceased breastfeeding, relactation by even half would reduce infant mortality arising from separation by nearly one third. Furthermore, researchers from Australia showed that early in the pandemic, unusually high numbers of mothers wanted support with relactation, being motivated to ensure food security and protect their infants from infection (39). It is notable that the number of guidance documents recommending wet nursing remained extremely low with only one in 20 recommending this practice. Prudhon et al. (40) identified that supporting relactation in emergencies is an area in need of further research and Smith and Iellamo (41) identified that an absence of guidance on wet nursing impedes support for this option in emergencies. The low uptake of recommendations for relactation and wet nursing confirms the need for further research and guidance.

Psychological support for mothers separated from their infants was the recommendation that most increased in frequency between March-April 2020 and November-December 2020 in the guidance documents collected for this research. In many respects this is not surprising since, as the pandemic progressed, it became clear that social isolation, physical inactivity, economic insecurity, and domestic violence, together with specific COVID-19 fears adversely impacted the mental health of pregnant women and new mothers (42–45). Mothers whose infants were separated from them because they had COVID-19, experienced greater distress as compared to women who were not separated (12, 46). However, although the number of country guidance recommending psychological support increased, it was still present in less than half of the guidance. This is troubling, not only because of the impact of separation without support on women, but also because of the potential impact of poor mental health on maternal caregiving and therefore on infant mental health and child development (47).

### 4.1. Psychological support for separated infants

Less than 10% of countries included a recommendation that infants separated from their mothers should be provided with psychological support. Maternal separation is distressing for infants. From birth, infants know the voice (48) and smell (49) of their mother and are distressed by their mothers’ absence (50). Separation from their mother and lack of comfort constitutes a significant psychological insult (51). Moreover, nursing staff may be unable to provide comfort to separated infants because of time constraints (52) and over the course of the pandemic, videos have emerged of infants in hospital nurseries alone and crying. Many forms of psychological support that are available to mothers (for example verbal reassurance and video calls) cannot assist infants. However, in the absence of the mother, others are able to provide comfort (53, 54). Thus, provision of an alternative caregiver is an appropriate intervention, as recommended in some of the guidance documents in this study. It is disturbing nonetheless that the psychological needs of infants separated from their mothers was recognized by so few countries’ guidance documents.

The impact of separation and interruption of breastfeeding on maternal attachment and caregiving capacity is known (9) and reduced maternal-infant attachment scores from separation due to COVID-19 have been documented (55). However, support for separated mothers to attach to their infants after reunification could assist. Skin-to-skin contact (56), infant massage (57), carrying (58), and breastfeeding (59) have all been shown to support the development of maternal-infant attachment. However, neither the WHO guidance nor any of the guidance included in this study provided specific recommendations on supporting separated mothers and infants after reunification. This is an oversight that must be rectified.

### 4.2. Influence of external guidance on country recommendations

In the guidance collected during March-April 2020, the USCDC was the most influential organization cited by guidance from 40% of the countries, followed by the RCOG (38%) and then the WHO (21%). In the November-December 2020 guidance collection, the WHO and RCOG were cited by guidance from 50% of the countries and the USCDC (inclusive of ACOG) by 39%. These data raise several questions. First, given that 99 out of 101 of the countries whose guidance was included in this study are members of the WHA, why was the WHO not more frequently cited and recommendations applied? In our first guidance collection, the relative infrequency with which the WHO was referenced could have partly been because the WHO guidance had been only recently published. However, the second guidance collection was initiated 8 months after WHO first published their COVID-19 clinical guidance, giving plenty of time for countries to assimilate WHO’s recommendations. In some cases, the WHO might not have been cited because countries had not updated their guidance after initial publication; nearly one third of countries’ most recent guidance was published during February-April 2020. However, the fact that guidance documents from 38 Ministries of Health in WHA member states did not reference guidance from the WHO is concerning.

Second, why was the USCDC still cited frequently even as evidence became overwhelming that maternal-infant proximity and breastfeeding was far safer than separation? As with the lack of use and citation of the WHO, early publication of country guidance documents and the lack of revision of some guidance documents may have contributed to these lapses. However, the work of the USCDC in global public health may have also encouraged Ministries of Health and professional associations to view the USCDC as a reliable authority whose recommendations could be applied in their own context. Within the USA, domestic health authorities, including the USCDC, worked together to ensure that their organizational recommendations did not conflict and cause confusion domestically (60). However, the experience of the COVID-19 pandemic suggests that the USCDC needs to also consider the influence of their recommendations internationally. In future pandemics, consideration should be given to reducing international confusion where USCDC recommendations conflict with the WHO risking causing harm. Greater coordination between the USCDC and the WHO may be warranted as well as indicating clearly when USCDC recommendations are intended only for domestic use. One can speculate that the effect of the COVID-19 pandemic on infant morbidity and mortality could have been lessened if the USCDC had been unified with the WHO and the RCOG in recommending maternal proximity and breastfeeding for mothers with COVID-19 from the beginning of the pandemic.

### 4.3. Limitations

It is a limitation of this study that there was no assessment of whether and how country guidance may have changed since collection. Guidance was not collected from all countries and it was assumed that guidance published on Ministry of Health websites was current when this may not have been the case. Where guidance was not dated, the date of publication could not be ascertained and where guidance did not contain references, it could not be determined how the guidance was influenced by other sources. Finally, the degree to which health professionals in hospitals followed guidance was not explored and should be considered in future research.

### 4.4. Conclusion

Our analysis of COVID-19 clinical guidance for maternal and newborn care from 101 countries showed that concerns regarding SARS-CoV-2 transmission through maternal proximity or breastfeeding took precedence over the evidence that impeding breastfeeding would be more harmful. Although there was improvement between country guidance gathered during March-April 2020 and November-December, COVID-19 maternal and newborn care guidance from most countries still failed to treat skin-to-skin contact, rooming-in and breastfeeding as the standard of care. In many country guidance documents, maternal proximity and breastfeeding were treated as an exception, sometimes requiring “informed consent” if allowed at all. Many health authorities also declined to provide clear recommendations even as evidence about COVID-19 and the safety of maternal proximity and breastfeeding grew, contrary to the principle of do no harm. While the influence of the WHO guidance increased, the USCDC remained influential globally and early recommendations for isolation of infants from their mothers persisted. This analysis has demonstrated that in the absence of quality evidence of necessity, recommendations against breastfeeding should not be made, particularly early in disease epidemics as these recommendations can persist despite evolving evidence to the contrary. Furthermore, international cooperation in the development and management of guidance is needed to ensure that recommendations made in one country do not undermine the advice of the WHO elsewhere. The COVID-19 pandemic has highlighted again that confidence in breastfeeding and its importance is fragile and the mother-infant relationship is undervalued. The value of breastfeeding in protecting against infectious and non-infectious disease, supporting maternal caregiving, providing food security and brain development must be more widely and deeply understood.

## Data availability statement

The original contributions presented in this study are included in the article/Supplementary material, further inquiries can be directed to the corresponding author.

## Author contributions

DV, KG, and KM conceived of the study. DV, KG, KM, and RM acquired guidance documents. DV, JC, KG, KM, and RM analyzed and interpreted data. All authors drafted and revised the manuscript, approved the final version, and agreed to be accountable for all aspects of the work.
