# Corrigendum: *First do no harm* overlooked: Analysis of COVID-19 clinical guidance for maternal and newborn care from 101 countries shows breastfeeding widely undermined

**DOI:** 10.3389/fnut.2023.1166221

**Published:** 2023-03-02

**Authors:** Karleen Gribble, Jennifer Cashin, Kathleen Marinelli, Duong Hoang Vu, Roger Mathisen

**Affiliations:** ^1^School of Nursing and Midwifery, Western Sydney University, Parramatta, NSW, Australia; ^2^Alive & Thrive Southeast Asia, FHI 360, Washington, DC, United States; ^3^Department of Pediatrics, University of Connecticut School of Medicine, Hartford, CT, United States; ^4^Alive & Thrive Southeast Asia, FHI 360, Hanoi, Vietnam

**Keywords:** COVID-19, breastfeeding, policy, psychosocial support systems, rooming-in care

In the published article, there was an error in Figure 6 as published. China and Mongolia were shaded white when they should have been shaded gray and counted among the countries with “no recommendation made.”

The corrected Figure 6 appears below.

**Figure 6:**
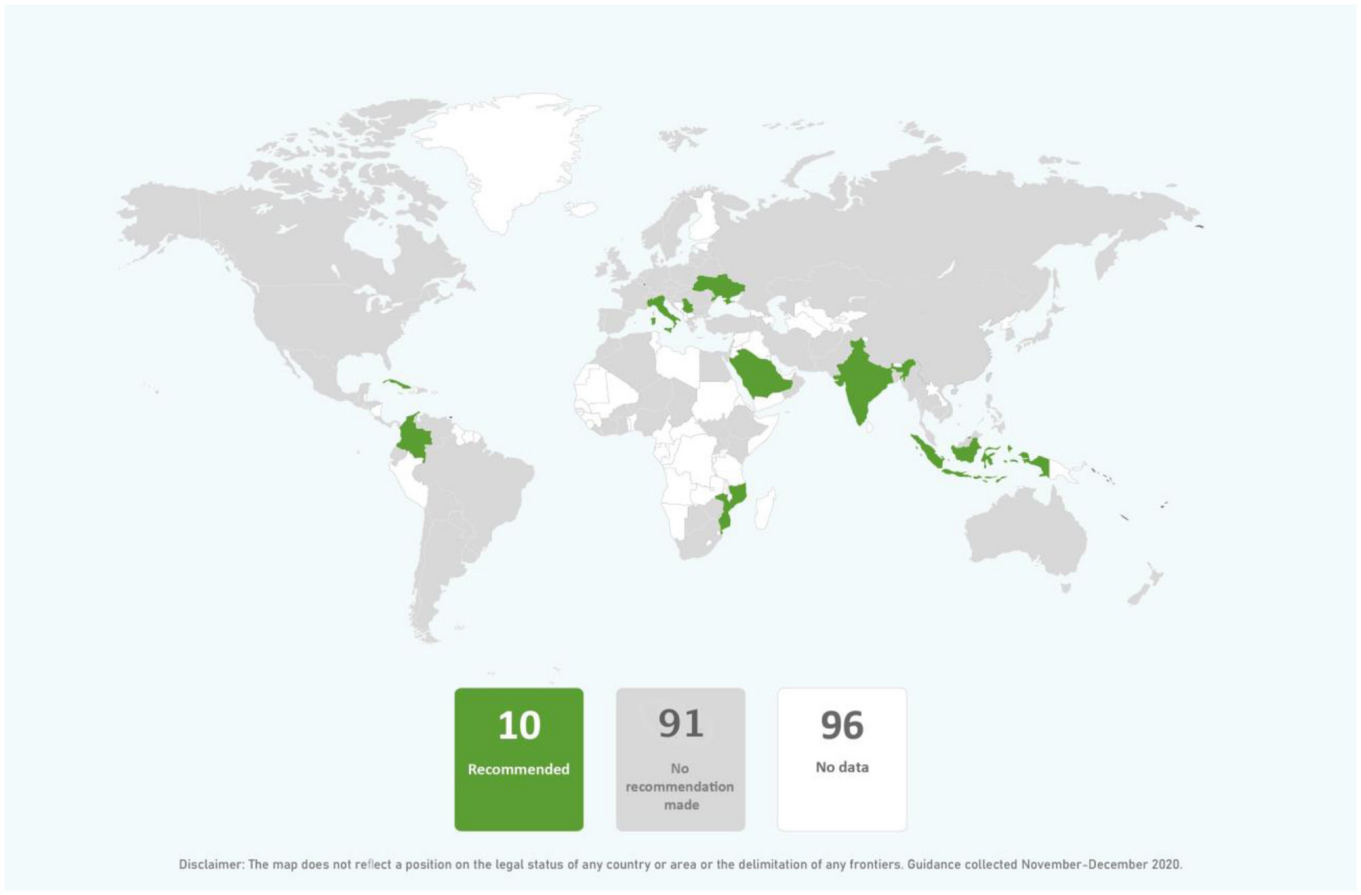
Global distribution of recommendations on relactation support for mothers with COVID-19 (data from 101 countries).

In the published article, there was an error in Figure 8. The 93 countries with “no recommendation made” should have been shaded gray instead of white.

The corrected Figure 8 appears below.

**Figure 8:**
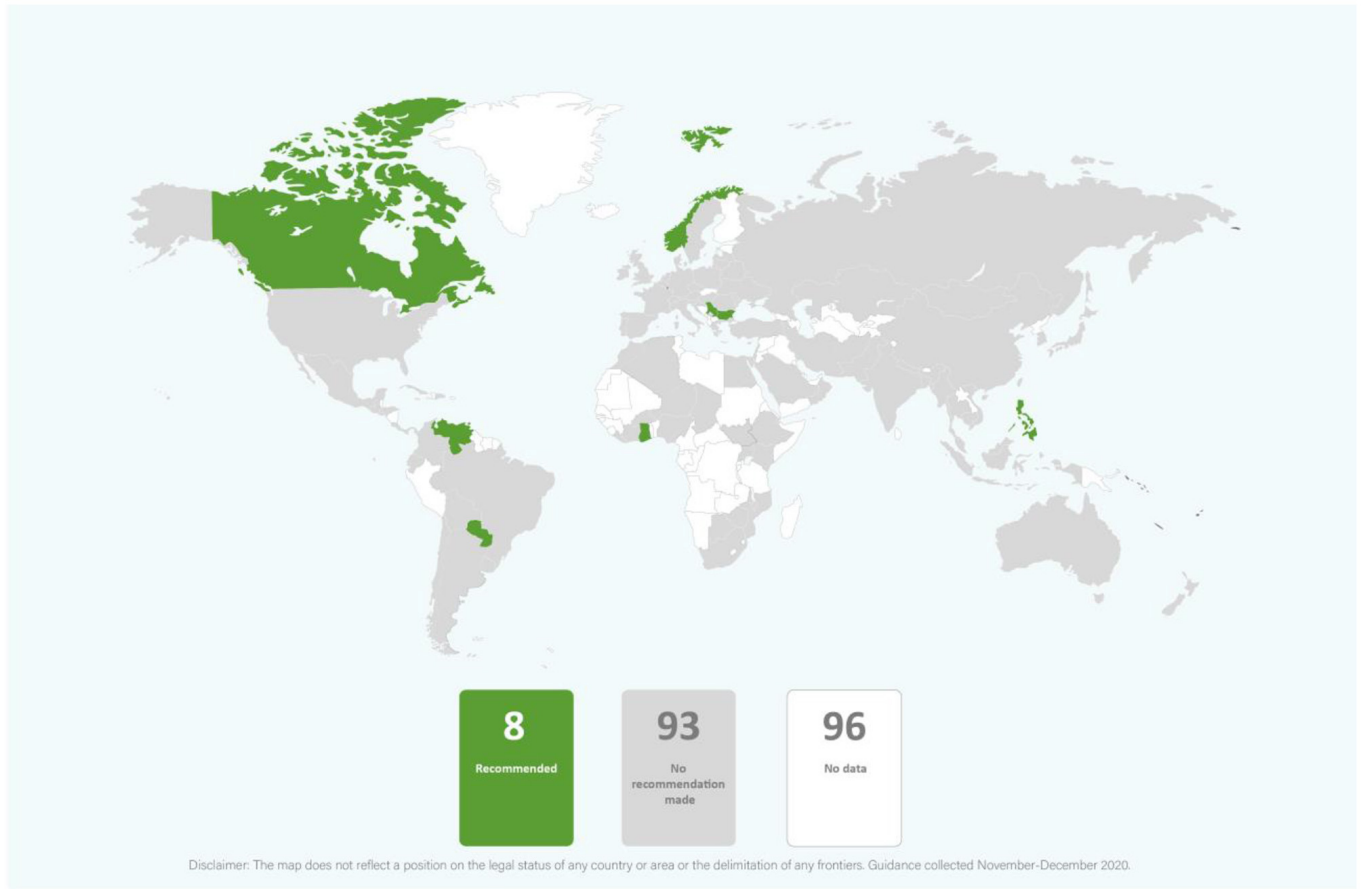
Global distribution of recommendations on psychological support for infants of mothers with COVID-19 (data from 101 countries).

In the published article, there was an error in Figure 11 as published. The name of the country “South Sudan” was in the image when it should not have been.

The corrected Figure 11 appears below.

**Figure 11:**
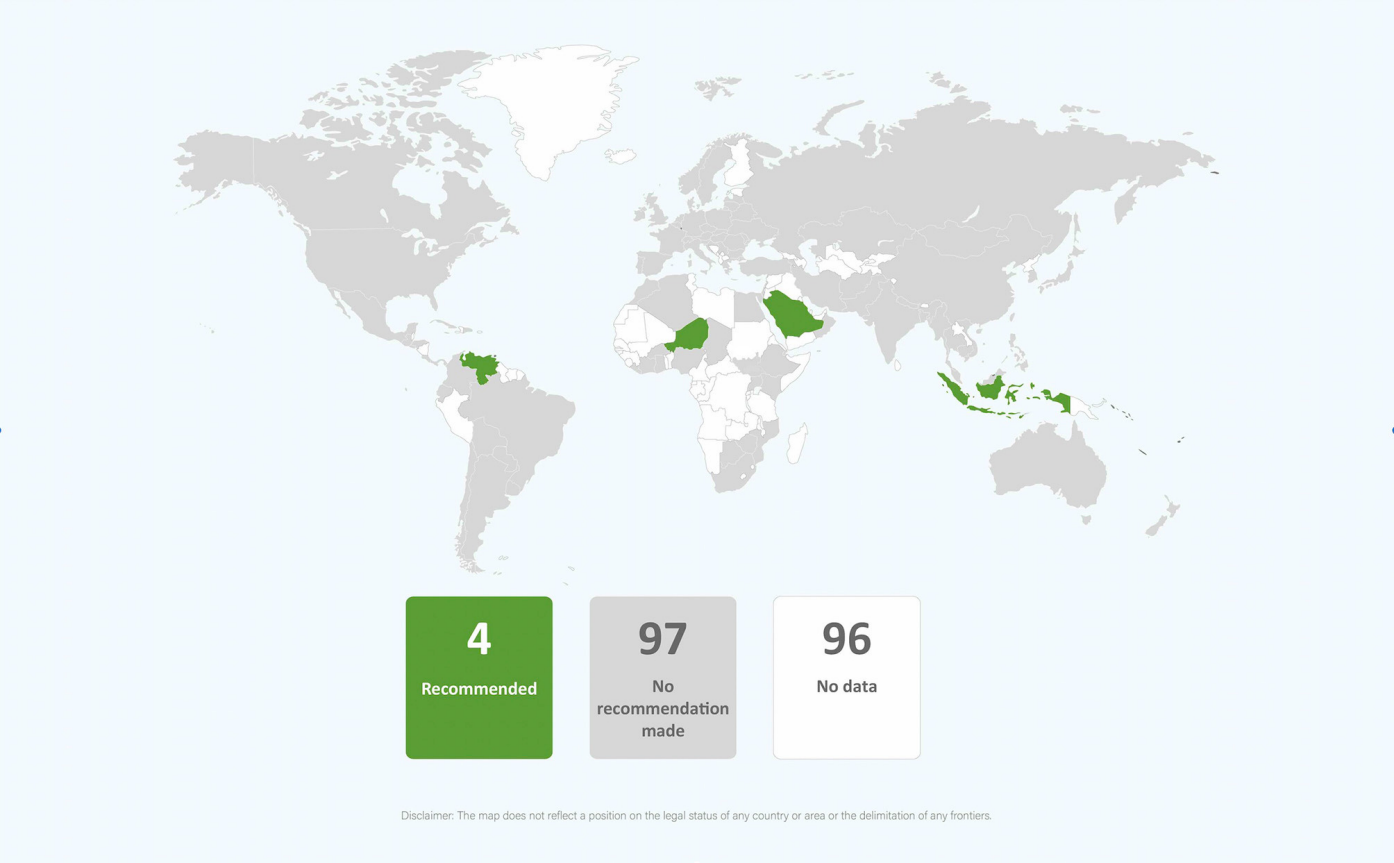
Global distribution of recommendations on wet nursing infants of mothers with COVID-19 (data from 101 countries).

The authors apologize for these errors and state that this does not change the scientific conclusions of the article in any way. The original article has been updated.

