# Supplementary material for: First do no harm overlooked: Analysis of COVID-19 clinical guidance for maternal and newborn care from 101 countries shows breastfeeding widely undermined
Source: Front Nutr. 2023 Jan 17;9:1049610. doi: 10.3389/fnut.2022.1049610 (PMC9889271; doi:10.3389/fnut.2022.1049610)
Supplement: Supplementary file 1 [file Data_Sheet_1.zip › Supplementary materials/2. Supplementary Figure 1. Month of publication of the most recently published guidance from each country.DOCX]

Supplementary Figure 1: Month of publication of the most recently published guidance from each country (N=95)
