# Supplementary material for: First do no harm overlooked: Analysis of COVID-19 clinical guidance for maternal and newborn care from 101 countries shows breastfeeding widely undermined
Source: Front Nutr. 2023 Jan 17;9:1049610. doi: 10.3389/fnut.2022.1049610 (PMC9889271; doi:10.3389/fnut.2022.1049610)
Supplement: Supplementary file 1 [file Data_Sheet_1.zip › Supplementary materials/3. Supplementary Figure 2. Change in alignment with eleven WHO COVID-19 recommendations for maternal and newborn care.DOCX]

Supplementary Figure 2: Change^1^ in alignment with eleven WHO COVID-19 recommendations for maternal and newborn care (N=32)

*^1^ From March-April 2020 to November-December 2020*
