# Supplementary material for: First do no harm overlooked: Analysis of COVID-19 clinical guidance for maternal and newborn care from 101 countries shows breastfeeding widely undermined
Source: Front Nutr. 2023 Jan 17;9:1049610. doi: 10.3389/fnut.2022.1049610 (PMC9889271; doi:10.3389/fnut.2022.1049610)
Supplement: Supplementary file 1 [file Data_Sheet_1.zip › Supplementary materials/4. Supplementary Table 2. Alignment of national COVID-19 guidance on breastfeeding and newborn care with WHO recommendations-our data.DOCX]

**Supplementary Table 2.** Alignment of national COVID-19 guidance on breastfeeding and newborn care with WHO recommendations: our data.

| Country | Income level | **S2S** | **EIBF** | **RI** | **BF** | **EBM** | **DHM** | **WN** | **BMS** | **PS-M** | **PS-I** | **RL** | **WHO alignment score** |
| --- | --- | --- | --- | --- | --- | --- | --- | --- | --- | --- | --- | --- | --- |
| Afghanistan | L | 0 | 0 | x | x | v | 0 | 0 | 0 | 0 | 0 | 0 | 1 |
| Algeria | L | 0 | 0 | x,2 | v | 0 | 0 | 0 | 0 | 0 | 0 | 0 | 1 |
| Argentina | UM | v | 0 | x,2,f | x,f | v | 0 | 0 | 0 | 0 | 0 | 0 | 2 |
| Armenia | UM | v | 0 | x,2,f | x,f | v | 0 | 0 | 0 | 0 | 0 | 0 | 2 |
| Australia | H | v | 0 | v | v | v | 0 | 0 | 0 | v | 0 | 0 | 5 |
| Austria | H | 0 | 0 | x,2,f | x,f | 0 | 0 | 0 | 0 | v | 0 | 0 | 1 |
| Bahrain | H | x | x | x,2,f | x,f | v | 0 | 0 | 0 | 0 | 0 | 0 | 1 |
| Bangladesh | LM | v | 0 | v | v | v | 0 | 0 | 0 | v | 0 | 0 | 5 |
| Belarus | UM | x | x | x | x | 0 | 0 | 0 | x | 0 | 0 | 0 | 0 |
| Belgium | H | v | 0 | x,2 | v | v | 0 | 0 | 0 | 0 | 0 | 0 | 3 |
| Bolivia | LM | x | x | x | x,f | v | 0 | 0 | x | 0 | 0 | 0 | 1 |
| Botswana | UM | 0 | 0 | v | v | v | 0 | 0 | 0 | 0 | 0 | 0 | 3 |
| Brazil | UM | 0 | 0 | x,2 | x,f | x,f | v | 0 | 0 | v | 0 | 0 | 2 |
| Bulgaria | UM | 0 | 0 | v | v | 0 | 0 | 0 | 0 | 0 | v | 0 | 3 |
| Burkina Faso | L | v | 0 | v | v | v | v | 0 | v | 0 | 0 | 0 | 6 |
| Cambodia | LM | v | v | v | v | v | 0 | 0 | 0 | v | 0 | 0 | 6 |
| Canada | H | 0 | 0 | v | v | v | 0 | 0 | 0 | v | v | 0 | 5 |
| Chad | L | 0 | 0 | x,2 | v | 0 | 0 | 0 | 0 | 0 | 0 | 0 | 1 |
| Chile | H | x,f | 0 | x,2,f | v | v | 0 | 0 | 0 | 0 | 0 | 0 | 2 |
| China | UM | x | x | x | x | x | 0 | 0 | 0 | 0 | 0 | 0 | 0 |
| Colombia | UM | x,f | x,f | x,2,f | x,f | v | v | 0 | v | 0 | 0 | v | 4 |
| Costa Rica | UM | v | 0 | x,2 | v | v | 0 | 0 | 0 | 0 | 0 | 0 | 3 |
| Côte d’lvoire | LM | x | x,f | 0 | v | v | 0 | 0 | v | 0 | 0 | 0 | 3 |
| Croatia | H | x | x | x | x | v | 0 | 0 | 0 | 0 | 0 | 0 | 1 |
| Cuba | UM | x | x | x | x | 0 | v | 0 | 0 | 0 | 0 | v | 2 |
| Cyprus | H | x | x | x,2,f | v | v | 0 | 0 | 0 | 0 | 0 | 0 | 2 |
| Czech Republic | H | 0 | 0 | x,2 | v | v | 0 | 0 | 0 | 0 | 0 | 0 | 2 |
| Denmark | H | v | 0 | v | v | v | 0 | 0 | 0 | 0 | 0 | 0 | 4 |
| Djbouti | LM | 0 | 0 | x,2 | v | 0 | 0 | 0 | 0 | 0 | 0 | 0 | 1 |
| Dominican Republic | UM | v | v | v | v | 0 | 0 | 0 | 0 | v | 0 | 0 | 5 |
| Ecuador | UM | v | v | v | v | v | v | 0 | 0 | 0 | 0 | 0 | 6 |
| Egypt | LM | v | v | x,2 | v | v | 0 | 0 | 0 | v | 0 | 0 | 5 |
| El Salvador | LM | x | 0 | x | v | v | v | 0 | v | 0 | 0 | 0 | 4 |
| Ethiopia | L | v | v | v | v | v | 0 | 0 | v | v | 0 | 0 | 7 |
| France | H | 0 | 0 | x,2 | v | 0 | 0 | 0 | 0 | v | 0 | 0 | 2 |
| Germany | H | v | 0 | x,2,f | v | v | 0 | 0 | 0 | 0 | 0 | 0 | 3 |
| Ghana | LM | v | v | v | v | v | 0 | 0 | v | v | v | 0 | 8 |
| Greece | H | 0 | 0 | x,2,f | x,f | v | 0 | 0 | 0 | 0 | 0 | 0 | 1 |
| Guatemala | UM | x | x | x,2 | x | v | 0 | 0 | 0 | 0 | 0 | 0 | 1 |
| Honduras | LM | x | v | v | v | v | 0 | 0 | 0 | 0 | 0 | 0 | 4 |
| Hungary | H | 0 | 0 | x,2 | v | v | 0 | 0 | 0 | 0 | 0 | 0 | 2 |
| India | LM | v | v | v | v | v | v | 0 | v | 0 | 0 | v | 8 |
| Indonesia | UM | 0 | x,f | x | x | v | v | v | v | v | 0 | v | 6 |
| Iran | UM | v | v | x,2 | v | v | v | 0 | v | 0 | 0 | 0 | 6 |
| Ireland | H | x | 0 | x,2 | v | v | 0 | 0 | 0 | v | 0 | 0 | 3 |
| Israel | H | x | 0 | x,2 | v | v | 0 | 0 | 0 | 0 | 0 | 0 | 2 |
| Italy | H | v | v | v | v | v | v | 0 | v | v | 0 | 0 | 9 |
| Jamaica | UM | 0 | 0 | v | x,f | v | 0 | 0 | v | 0 | 0 | 0 | 3 |
| Japan | H | x | x | x | x | x | 0 | 0 | x | 0 | 0 | 0 | 0 |
| Kazakhstan | UM | x | x | x | x | x | 0 | 0 | 0 | v | 0 | 0 | 1 |
| Kenya | LM | x | x | x,f | x,f | v | v | 0 | v | v | 0 | 0 | 4 |
| Kosovo | UM | v | v | v | v | v | 0 | 0 | 0 | v | 0 | 0 | 6 |
| Latvia | H | 0 | 0 | x | x | x,f | 0 | 0 | 0 | 0 | 0 | 0 | 0 |
| Lebanon | UM | x | 0 | x,2 | x,f | v | 0 | 0 | 0 | 0 | 0 | 0 | 1 |
| Liberia | L | x | v | x,2 | x | v | 0 | 0 | v | v | 0 | 0 | 4 |
| Lithuania | H | x | x | x | x | v | 0 | 0 | v | 0 | 0 | 0 | 2 |
| Malaysia | UM | x,f | 0 | x,2,f | x | x,f | 0 | 0 | v | v | 0 | 0 | 2 |
| Mexico | UM | v | v | x,2 | v | v | v | 0 | v | v | 0 | 0 | 7 |
| Moldova | LM | v | v | v | v | v | 0 | 0 | 0 | v | 0 | 0 | 6 |
| Mongolia | LM | 0 | 0 | 0 | x | v | 0 | 0 | 0 | v | 0 | 0 | 2 |
| Morocco | LM | 0 | v | v | v | v | 0 | 0 | 0 | v | 0 | 0 | 5 |
| Mozambique | L | v | v | v | v | v | 0 | 0 | v | 0 | 0 | v | 7 |
| Myanmar | LM | 0 | 0 | v | x,f | v | 0 | 0 | 0 | 0 | 0 | 0 | 2 |
| Nepal | LM | v | v | v | v | v | 0 | 0 | 0 | v | 0 | 0 | 6 |
| Netherlands | H | 0 | 0 | 0 | v | 0 | 0 | 0 | 0 | 0 | 0 | 0 | 1 |
| New Zealand | H | v | 0 | x,2 | v | 0 | 0 | 0 | 0 | 0 | 0 | 0 | 2 |
| Niger | L | v | v | v | v | v | 0 | v | v | v | 0 | 0 | 8 |
| Nigeria | LM | v | v | 0 | v | 0 | 0 | 0 | 0 | 0 | 0 | 0 | 3 |
| Norway | H | v | v | v | v | v | v | 0 | v | v | v | 0 | 9 |
| Oman | H | x | 0 | 0 | x,f | v | 0 | 0 | 0 | 0 | 0 | 0 | 1 |
| Pakistan | LM | v | 0 | 0 | v | 0 | 0 | 0 | 0 | 0 | 0 | 0 | 2 |
| Panama | H | x | x | 0 | x,f | v | 0 | 0 | 0 | 0 | 0 | 0 | 1 |
| Paraguay | UM | x,f | v | x,2,f | v | v | v | 0 | v | 0 | v | 0 | 6 |
| Philippines | LM | 0 | v | x,2,f | v | v | v | 0 | v | v | v | 0 | 7 |
| Poland | H | x,f | x,f | x,f | x,f | v | 0 | 0 | 0 | 0 | 0 | 0 | 1 |
| Portugal | H | x,f | 0 | x,2,f | x,f | x,f | x | 0 | 0 | v | 0 | 0 | 1 |
| Romania | H | x,f | 0 | x,f | 0 | v | 0 | 0 | x | 0 | 0 | 0 | 1 |
| Russia | UM | x | x | x | x | v | 0 | 0 | 0 | 0 | 0 | 0 | 1 |
| Saudi Arabia | H | x | x | x | x | v | v | v | v | v | 0 | v | 6 |
| Serbia | UM | v | v | x | v | v | 0 | 0 | v | 0 | v | v | 7 |
| Singapore | H | x | x | x | x | x | 0 | 0 | 0 | 0 | 0 | 0 | 0 |
| Slovakia | H | x | 0 | x,2,f | x,f | x,f | x | 0 | x | 0 | 0 | 0 | 0 |
| Slovenia | H | 0 | 0 | v | v | v | 0 | 0 | 0 | v | 0 | 0 | 4 |
| South Africa | UM | v | v | v | v | v | v | 0 | 0 | v | 0 | 0 | 7 |
| South Korea | H | x | x | x,2,f | x | x | 0 | 0 | x | 0 | 0 | 0 | 0 |
| South Sudan | L | 0 | 0 | v | v | v | 0 | 0 | x | v | 0 | 0 | 4 |
| Spain | H | v | v | x,2 | v | v | v | 0 | 0 | 0 | 0 | 0 | 5 |
| Sweden | H | 0 | 0 | v | v | v | 0 | 0 | 0 | 0 | 0 | 0 | 3 |
| Switzerland | H | 0 | 0 | x,f | v | 0 | 0 | 0 | 0 | 0 | 0 | 0 | 1 |
| Taiwan | H | x | x | x | x | 0 | 0 | 0 | 0 | 0 | 0 | 0 | 0 |
| Thailand | UM | 0 | 0 | 0 | x,f | x,f | 0 | 0 | 0 | 0 | 0 | 0 | 0 |
| Timor Leste | LM | v | v | v | v | v | v | 0 | 0 | v | 0 | v | 8 |
| Turkey | UM | x | x | x | v | v | 0 | 0 | 0 | 0 | 0 | 0 | 2 |
| Uganda | L | 0 | 0 | v | v | v | 0 | 0 | 0 | v | 0 | 0 | 4 |
| Ukraine | LM | 0 | 0 | v | v | v | v | 0 | 0 | 0 | 0 | v | 5 |
| United Kingdom | H | v | v | v | v | v | v | 0 | 0 | v | 0 | 0 | 7 |
| United States of America | H | 0 | 0 | x,2,f | 0 | 0 | 0 | 0 | 0 | v | 0 | 0 | 1 |
| Uruguay | H | x,f | 0 | 0 | v | v | 0 | 0 | 0 | 0 | 0 | 0 | 2 |
| Venezuela | 0 | v | v | v | v | v | 0 | v | 0 | v | v | 0 | 8 |
| Vietnam | LM | 0 | 0 | x | x | v | v | 0 | v | 0 | 0 | 0 | 3 |
| Zimbabwe | LM | 0 | 0 | 0 | v | v | 0 | 0 | 0 | 0 | 0 | 0 | 2 |
| **International Organization** | **Income level** | **S2S** | **EIBF** | **RI** | **BF** | **EBM** | **DHM** | **WN** | **BMS** | **PS-M** | **PS-I** | **RL** | **WHO alignment score** |
| Pacific Joint Incident Management Team | 0 | v | v | v | v | v | 0 | 0 | 0 | v | 0 | 0 | 6 |
| Pan American Health Organization | 0 | v | v | v | v | v | v | v | v | v | v | v | 11 |

*Note*. Income level = World Bank levels L= low-income, LM = lower-middle income, UM = upper-middle income, H = high-income. Variables= S2S = Skin-to-skin contact; EIBF = Early Initiation of Breastfeeding; RI = Rooming-in; BF = Direct breastfeeding with infection protection and control measures; EBM = Expressed milk recommended if direct breastfeeding not available; DHM = Donor Human Milk recommended if maternal milk not available; WN = Wet nursing recommended if maternal milk not available; BMS = Breastmilk substitutes recommended if breastmilk not available; PS-M = psychological support for mothers where separated; PS-I = psychological support for infants where separated; RL = Relactation; WHO alignment score = Number of recommendations aligned with WHO (out of 11);

Coding: 0=No recommendation made; v = Recommended; x = Not Recommended; x, f = Allowed based on family preference; x, 2 = Room sharing allowed with two meters distance; x, 2, f = Room sharing allowed with two meters distance upon family request/preference or decision of health professionals.
