# Supplementary material for: First do no harm overlooked: Analysis of COVID-19 clinical guidance for maternal and newborn care from 101 countries shows breastfeeding widely undermined
Source: Front Nutr. 2023 Jan 17;9:1049610. doi: 10.3389/fnut.2022.1049610 (PMC9889271; doi:10.3389/fnut.2022.1049610)
Supplement: Supplementary file 1 [file Data_Sheet_1.zip › Supplementary materials/1. Supplementary Table 1. Guidance documents included in the review (N=125) from 101 countries and 2 international organisations.docx]

**Supplementary Table 1.** Guidance documents included in the review (*N*=125) from 101 countries and 2 international organizations

| **Country of origin** | **Title of document (translated)** | **Type ^a^** | **Date released (2020)** | **Source ^b^** |
| --- | --- | --- | --- | --- |
| Afghanistan | Government of the Islamic Republic of Afghanistan Ministry of Public Health, Protocol for the Care of Pregnant Women Suspected or Confirmed with COVID-19 | GG | June 25 | <https://moph.gov.af/sites/default/files/2020-06/%D9%BE%D8%B1%D9%88%D8%AA%D9%88%DA%A9%D9%88%D9%84%20%D9%85%D8%B1%D8%A7%D9%82%D8%A8%D8%AA%20%D9%87%D8%A7%DB%8C%20%DA%A9%D9%88%DB%8C%D8%AF-19%20%D9%86%D8%B2%D8%AF%20%D8%AE%D8%A7%D9%86%D9%85%20%D9%87%D8%A7%DB%8C%20%D8%AD%D8%A7%D9%85%D9%84%D9%87.pdf> |
| Algeria | Democratic People's Republic of Algeria Ministry of Health and the Reform of Hospitals, Project for the Taking Care of the Pregnant Woman with COVID-19 | GG | May 4 | <http://www.sante.gov.dz/images/Prevention/cornavirus/InstructionCovid/Instruc-12-Parturiente.PDF> ^c^ |
| Argentina | Ministry of Health Argentina, Recommendations for the Care of Pregnant and Newborn in Relation to COVID-19 | GG | July 20 | <https://bancos.salud.gob.ar/sites/default/files/2020-07/covid-19-recomendaciones-atencion-embarazadas-recien-nacidos.pdf> |
| Armenia | Order of the Minister of Health of the Republic of Armenia, New Coronavirus Infection in Armenia (2019 N-COV) Confirmed or Suspected in Children, Pregnant Women and Newborns of Mothers with Confirmed or Suspected COVID-19 | GG | May 12 | <https://ncdc.am/coronavirus/technical-documents/> |
| Australia  (Victoria) | Maternity and neonatal care during coronavirus (COVID-19)  Coronavirus (COVID-19) update | GG | October 16 | <https://www.dhhs.vic.gov.au/covid-19-maternity-and-neonatal-care-during-coronavirus> ^c^ |
| Austria | Guide to dealing with COVID-19 during pregnancy and the puerperium: Information from the Austrian Society for Gynecology and Obstetrics (OEGGG) | PG | May 13 | <https://www.oeggg.at/app/download/9665261586/OEGGG-Leitfaden%20COVID-19-update-PK-2020-05-13-final.pdf?t=1604926524> |
| Bahrain | Bahrain National Taskforce for the Combatting the Coronavirus COVID-19, COVID-19 National Protocols | GG | October 13 |  |
| Bangladesh | National Guidelines on Clinical Management of  COVID-19 (Version 8.0) | GG | November 5 | <https://covidlawlab.org/wp-content/uploads/2021/01/Bangladesh_2020.11.05_Guideline_National-Guidelines-on-Clinical-Management-of-COVID-19_EN.pdf> |
| Belarus | Republic of Belarus Ministry of Health, Instructions on the Management of Patients from 0-18 years old with Confirmed COVID-19 Infection | GG | April 6 |  |
| Belgium | Belgium Pediatric COVID-19 Task force, Guideline for Newborn of a COVID-19 Positive Mother | PG | March 21 |  |
| Bolivia | Bolivian Society of Obstetrics and Gynecology, Protocol for the Management of the Pregnant Woman with Coronavirus (COVID-19) | PG/GG | May | <https://www.minsalud.gob.bo/component/jdownloads/?task=download.send&id=444&catid=30&m=0&Itemid=646> |
| Botswana | Botswana COVID-19 Guideline 9: Interim Clinical Guidance for the management of Paediatric patients with Coronavirus Disease 2019 (COVID-19) in Botswana | GG | May | <https://covid19portal.gov.bw/sites/default/files/2020-05/COVID-19-in-Paediatrics-Guideline.docx.pdf> |
| Brazil | Government of Brazil Ministry of Health, Manual of Recommendations for the Care of Pregnant and Puerperal Women in the Covid-19 Pandemic | GG | September 30 | <http://antigo.saude.gov.br/images/pdf/2020/September/02/Manual-de-Recomenda----es-para-Gestante.pdf> |
| Bulgaria | Bulgarian Neonatology Society, COVID-19 Guidelines for Neonatology Units | PG | March 19 | <https://nursing-bg.com/docs/covid19/8/12.pdf> |
| Burkina Faso | Society of Obstetricians and Gynecologists of Canada, Committee Opinion # 400: COVID-19 in Pregnancy | PG | July 27 | <https://sogob.org/?https-www-sogc-org-fr> ^c^ |
| Cambodia | Kingdom of Cambodia Ministry of Health, National COVID-19 Technical Brief for Maternal and Child Health Services | GG | 2020 |  |
| Canada | Government of Canada, Clinical Management of Patients with COVID-19: Second Interim Guidance | GG | August 17 |  |
| Chad | Republic of Chad Ministry of Public Health, COVID-19 Scientific Committee Protocols | GG | May 17 |  |
| Chile | Government of Chile Ministry of Health, Instructions for the Management of SARS-CoV-2 Cases (COVID-19) in Pregnancy and the Puerperium | GG | June | <http://www.colegiomedico.cl/wp-content/uploads/2020/08/Orientaciones-puerperas-gestantes-diadas.pdf> |
| China | Notice on strengthening the treatment and safe delivery of maternal diseases during the prevention and control of new coronavirus pneumonia | GG | February 8 | <http://www.nhc.gov.cn/xcs/zhengcwj/202002/4f80657b346e4d6ba76e2cfc3888c630.shtml> |
| Colombia | Colombia Ministry of Health, Interim guidelines for the health care of pregnant women, newborns and for breastfeeding in the context of pandemic COVID-19 in Colombia | GG | June | <https://www.minsalud.gov.co/Ministerio/Institucional/Procesos%20y%20procedimientos/GIPS14.pdf> |
| Costa Rica (1) | General Breastfeeding Guidelines for Mothers and COVID-19 | GG | March 8 | <https://www.ministeriodesalud.go.cr/sobre_ministerio/prensa/docs/lactancia_materna_v1_19032020.pdf> |
| Costa Rica (2) | Ministry of Health Costa Rica, Care Guideline During Pregnant, Childbirth, and Postpartum to Pregnant Women Infected by COVID-19 and the Newborn | GG | November 19 |  |
| Cote d'lvoire | Government of Cote d'lovire National Mother and Child Health Programme, Recommendations for Treatment in Obstetrics, Gynecology, and Neonatology During Coronavirus Pandemic Infection | GG | March 30 |  |
| Croatia | Croatian Society for Gynecology and Obstetrics, Coronavirus infection in pregnancy S2K guidelines for dealing with suspicion / coronavirus infections in pregnancy | PG | N.D. | <https://www.hdgo.hr/userFiles/upload/S2K-Smjernice-HDGO-Koronavirusna-Infekcija-u-Trudno%C4%87i.pdf> |
| Cuba | Cuba Ministry of Public Health, National Action Protocol for COVID-19 | GG | May | <https://files.sld.cu/editorhome/files/2020/05/MINSAP_Protocolo-de-Actuaci%C3%B3n-Nacional-para-la-COVID-19_versi%C3%B3n-1.4_mayo-2020.pdf> ^c^ |
| Cyprus | Nicosia Hospital, Recommendations for Care of a Newborn of a Mother with Confirmed or Suspected COVID-19 Disease |  | N.D. |  |
| Czech Republic | Neonatological Society of the Czech Medical Association, Recommendations for the Care of the Newborn and Breastfeeding in Quarantined and SARS-CoV-2 Positive Mothers | PG | April 2 | <http://www.porodnice.cz/clanky/doporuceni-neonatologicke-spolecnosti> |
| Denmark | Midwives' Association and Danish Society of Obstetrics and Gynaecology, Management of COVID-19 Infection in Pregnant and Birthing Women, their Partner, and the Newborn Baby | PG | July 8 | <https://static1.squarespace.com/static/5467abcce4b056d72594db79/t/5f073703406e70280290472d/1594308356788/Klinisk+vejledning+vedr%C3%B8rende+h%C3%A5ndtering+af+COVID-19_gravide+f%C3%B8dende+og+barslende+kvinder_version+7.pdf> |
| Djibouti | Republic of Djibouti Ministry of Health Action Plan for the Prevention and Response to COVID-19 Disease | GG | March 18 | <https://sante.gouv.dj/storage/publications/April2020/cMcQEVZc9fS4wLjvUSqA.pdf> |
| Dominican Republic | Dominican Republic Ministry of Public Health Guidelines for Prevention and Management of Respiratory Disease by Coronavirus (COVID-19) in Pregnant and Newborn | GG | April | <https://covid19-evidence.paho.org/handle/20.500.12663/1360?locale-attribute=pt_BR> |
| Ecuador | Ecuador Ministry of Public Health, Recommendations for prevention control and management of neonates with suspicion of SARS infection CoV-2 or confirmation by COVID-19 | GG | June | <https://www.salud.gob.ec/wp-content/uploads/2020/03/PROTOCOLO-NEONATALES-FIRMADO.pdf> |
| Egypt | Egypt Ministry of Health and Population, Management Guidelines for COVID-19 Patients with Special Medical Conditions | GG | April |  |
| El Salvador | Government of El Salvador Ministry of Health, Technical guidelines for care during pregnancy, childbirth puerperium and newborn for the COVID-19 emergency | GG | April | <http://asp.salud.gob.sv/regulacion/pdf/lineamientos/lineamientostecnicosparalaatencionduranteelembarazoelpartopuerperioydelreciennacidoporlaemergenciaporCOVID19-Acuerdo802.pdf> |
| Ethiopia | Ministry of Health Ethiopia, National Comprehensive COVID-19 Management Handbook | GG | April | <http://www.moh.gov.et/ejcc/sites/default/files/2020-06/NATIONAL%20COMPREHENSIVE%20COVID19%20MANAGEMENT%20HANDBOOK%20FIRST%20EDITION.pdf> |
| France (2) | College Nationale Des Gynecologues et Obstetriciens Francais, Protocol for Managing Contact, Possible or Confirmed Cases | PG | September 30 | <https://www.reseau-naissance.fr/medias/2020/11/COVID19_CNGOF_maj30092020.pdf> |
| Germany (1) | German Coalition of Obstetric and Pediatric Societies, Recommendations on SARS-CoV-2/COVID-19 in Pregnancy, Childbirth and the Puerperium | PG | October | https://web.archive.org/web/20201207021855/https://www.dggg.de/fileadmin/documents/Weitere_Nachrichten/2020/Update_COVID_Empfehlungen.pdf ^c^ |
| Germany (2) | Statement of the DGPI in agreement with the DGGG and the DGPM: Handling of Newborns of SARS-CoV-2 positive mothers | PG | June 30 | <https://dgpi.de/stellungnahme-dgpi-dggg-dgpm-umgang-mit-neugeborenen-sars-cov-2-positiver-muetter/> |
| Ghana | Maternal, Child Health and Nutrition Service Delivery Guidelines For Use In Ghana During Covid – 19 Outbreak | GG | April |  |
|  | Maternal Nutrition, And Infant & Young Child Feeding in The Context Of Covid-19 For Ghana | GG | March |  |
| Greece | Greek National Organisation of Public Health, Instructions for managing pregnant women with possible or confirmed COVID-19 infection | GG | March 25 | <https://eody.gov.gr/odigies-gia-ti-diacheirisi-egkyon-me-pithani-i-epivevaiomeni-loimoxi-covid-19/?fbclid=IwAR1Wp3ZsJgwRofcoNOh8eKxuCFBCvOsrQBcyCUivlve2EMGoJsa8g1JVBV0> |
| Guatemala | Guatemalan Institute of Social Security, Diagnosis and Patient Management Protocol Suspicious and Confirmed COVID-19 Obstetrics and Gynecology Hospital | GG | September 22 | <https://www.igssgt.org/wp-content/uploads/2020/09/Protocolo-de-diagnostico-t-manejo-de-pacientes-suspechosas-y-confirmadas-de-covid-19.pdf> |
| Honduras | Government of the Republic of Honduras Secretary of Health, Guidelines for the Care of Pregnant Women at Work, Delivery, and Puerperium, in the Context of COVID-19 | GG | March | <http://www.salud.gob.hn/site/index.php/component/edocman/lineamiento-atencion-de-embarazadas-y-covid19> |
| Hungary | Recommendation of Neonatal Department of Medical Professional College Guideline for the Management in Relation to the COVID 19 Pandemic | PG | March 21 | <https://webcache.googleusercontent.com/search?q=cache:q2mv6l2Uu_gJ:https://kollegium.aeek.hu/Download/Download/3452+&cd=2&hl=en&ct=clnk&gl=au> ^c^ |
| India | Government of India Ministry of Health and Family Welfare, Guidance Note on Provision of Reproductive, Maternal, Newborn, Child, Adolescent Health Plus Nutrition | GG | May 24 |  |
| Indonesia (1) | Republic of Indonesia Ministry of Health, Management Protocol COVID-19 (COVID-19 in Neonates) | GG | September 30 |  |
| Indonesia (2) | Indonesian Ministry of Health. Directorate General Public Health, Guidelines for Antenatal, Childbirth, Postpartum and Infant Care Newborn in the Age of Adaptation to New Habits | GG | September 16 |  |
| Iran (1) | Islamic Republic of Iran Ministry of Health and Medical Education, Newborn Care Guide from a Suspected or Confirmed Mother with COVID-19 Disease | GG | December |  |
| Iran (2) | Guide to Diagnosis and Treatment of COVID-19 in Pregnancy | GG | November |  |
| Ireland | Institute of Obstetricians and Gynaecologists, COVID-19 Infection Guidance for Maternity Services | GG | May 5 |  |
| Israel | The guidelines of the Society for Maternal and Fetal Medicine and the Israeli Association of Obstetrics and Gynecology regarding COVID-19 infection in pregnancy | PG | April 26 |  |
| Italy | Italian National Institute of Health, Interim Indications for Pregnancy, Childbirth, Breastfeeding and Care of the Very Young of 0-2 years in Response to the COVID-19 emergency | GG | May 31 | <https://www.iss.it/rapporti-covid-19/-/asset_publisher/btw1J82wtYzH/content/id/5421215?_com_liferay_asset_publisher_web_portlet_AssetPublisherPortlet_INSTANCE_btw1J82wtYzH_redirect=https%3A%2F%2Fwww.iss.it%2Frapporti-covid-19%3Fp_p_id%3Dcom_liferay_asset_publisher_web_portlet_AssetPublisherPortlet_INSTANCE_btw1J82wtYzH%26p_p_lifecycle%3D0%26p_p_state%3Dnormal%26p_p_mode%3Dview%26_com_liferay_asset_publisher_web_portlet_AssetPublisherPortlet_INSTANCE_btw1J82wtYzH_cur%3D0%26p_r_p_resetCur%3Dfalse%26_com_liferay_asset_publisher_web_portlet_AssetPublisherPortlet_INSTANCE_btw1J82wtYzH_assetEntryId%3D5421215> |
| Jamaica | Jamaica Ministry of Health and Wellness, Guideline for the Management of Pregnancy During the COVID-19 Pandemic | GG | March 25 | <https://www.moh.gov.jm/wp-content/uploads/2020/04/Guideline-for-the-Management-of-Pregnancy-During-the-COVID-19-Pandemic.pdf> |
| Japan | Japan Society of Obstetrics and Gynecology, the Japan Society of Obstetricians and Gynecologists, and the Japan Society of Obstetrics and Gynecology Infectious Diseases, Response to the new coronavirus infection (COVID-19) Guidelines for medical professionals 5th edition (for medical professionals) | PG | September 2 | <http://jsidog.kenkyuukai.jp/information/information_detail.asp?id=105938&fbclid=IwAR328GPqxnW97p5FKSzUXmEvRPMH3L6Jlg4KASch79wyxKiYXJEdp8ymLQc> |
| Kazakhstan | Republic of Kazakhstan Ministry of Health, Clinical Protocol Diagnostics and Treatment Coronavirus Infection COVID-19 | GG | July 4 | <https://www.gov.kz/uploads/2020/7/5/850cbfa66ca9360e9471952ff1c8de42_original.2720147.pdf> |
| Kenya (1) | Government of Kenya Ministry of Health, Guidelines on the Management of Paediatric Patients During COVID-19 Pandemic | GG | March 25 | <https://www.health.go.ke/wp-content/uploads/2020/06/PAEDIATRIC-Covid-Guidelines-Final.pdf> |
| Kenya (2) | Government of Kenya Ministry of Health, A Kenya Practical Guide for Continuity of Reproductive Maternal, Newborn and Family Planning Care and Service in the Background of COVID-19 Pandemic | GG | April | <https://www.health.go.ke/wp-content/uploads/2020/04/KENYA-COVID19-RMNH.pdf.pdf.pdf> |
| Kosovo | Republic of Kosovo Ministry of Health, Manual for COVID-19 Prevention and Control | GG | June | <https://msh.rks-gov.net/wp-content/uploads/2020/06/Manual-p%C3%ABr-parandalimindhe-luftimin-e-COVID-19-ENG.docx> |
| Latvia | Association of Gynecologists and Maternity Specialists, Recommendations for the Prevention and Control of COVID-19 Infection in a Maternity Hospital if a Pregnant Woman is Infected or Suspected of Being Infected with COVID-19 | PG | March 21 | <https://www.ginasoc.lv/search?s=Covid> |
| Lebanon | Lebanon Technical Taskforce of Corona in Pregnancy COVID-19 Virus Infection and Pregnancy, Labor & Delivery COVID-19 Guidelines | GG | April | <https://moph.gov.lb/userfiles/files/HealthCareSystem/Mother%26ChildHealth/Protocols%20of%20the%20Treatment/FV_%20Eng_%20INPATIENT%20COVID-19%20%20%20%20%20%20Guidelines.pdf> |
| Liberia | Ministry of Health and National Public Health Institute of Liberia, Interim Guidance on Clinical Care for Patients with COVID-19 in Liberia | GG | June 1 | <http://moh.gov.lr/wp-content/uploads/Interim_Guidance_for_care_of_Pts_with_Covid_19_in_Liberia.pdf> |
| Lithuania (1) | Lithuanian Society of Obstetricians and Gynecologists, COVID-19 Infection in Pregnancy | PG | September 24 | <https://sam.lrv.lt/uploads/sam/documents/files/COVID-19%20infekcija%20ir%20nestumas_Atnaujinta%202020%2009%2018.pdf> |
| Lithuania (2) | Lithuanian Neonatology Association, Recommendations on neonatal care, breastfeeding and COVID-19 infection | PG | March 18 | <https://www.lagd.lt/lietuvos-neonatologijos-asociacijos-rekomendacijos-del-naujagimio-prieziuros-zindymo-ir-covid-19-infekcijos/155> |
| Malaysia (1) | Malaysian Ministry of Health, Guidelines COVID-19 Management –Annex 23 (Pregnancy) | GG | March 31 | <http://covid-19.moh.gov.my/garis-panduan/garis-panduan-kkm/Annex_23_Guidelines_COVID_Pregnancy_22032020.pdf?fbclid=IwAR22IEJSn12G-nmf2jojHkexs4d1hzyRPzoY577mo_YghTZRZt2wItd01NU> |
| Malaysia (2) | Malaysian Ministry of Health, Guidelines COVID-19 Management –Annex 31 (Newborn) | GG | December 5 | <http://covid-19.moh.gov.my/garis-panduan/garis-panduan-kkm/Annex_31_Management_COVID_19_in_Neonates.pdf> |
| Mexico (1) | Government of Mexico, Guidelines for the prevention and mitigation of COVID-19 in pregnancy and the puerperium | GG | July 20 | <https://coronavirus.gob.mx/wp-content/uploads/2020/07/Lineamientos_Prevencion_COVID19_Embarazos_V2.pdf?fbclid=IwAR1J1g1njWOTp2IkIYUkLitH326pbjfXfhmS4rfNcnP3pg7Z61zhjBt5vRI> |
| Mexico (2) | Government of Mexico, Addressing COVID-19 in Newborns | GG | October 7 | <https://coronavirus.gob.mx/wp-content/uploads/2020/10/COVID_RN_Hoja_actualizacion.pdf?fbclid=IwAR1CHRGOLgx-3dW7ezI2wTee2ilLwBsMD9Ir1ro2wyJyqynvILk2JRp0f-0> |
| Moldova | Government of Moldova Ministry of Health, Labor and Social Protection, COVID-19 National Clinical Protocol | GG | March 30 | <https://msmps.gov.md/sites/default/files/pcn_provizoriu_infectia_cu_coronavirus_de_tip_nou_covid-19_aprobat_prin_ordinul_msmps_nr.336_din_30.03.2020_ru.pdf> ^c^ |
| Mongolia | Mongolia COVID-19 Handbook | GG | ND |  |
| Morocco | Organization and support pregnancy, childbirth and postpartum during the COVID-19 pandemic period: Practical guide for use by health professionals | GG | May |  |
| Mozambique | Ministry of Health, Recommendations for Infant Feeding in the Event of Suspected or Confirmed COVID-19: Best Practices Pack | GG | May |  |
| Myanmar | Government of the Republic of the Union of Myanmar Ministry of Health and Sports, Clinical Management Guideline for COVID-19 Infection in Pregnancy | GG | April 9 |  |
| Nepal | Federal Democratic Republic of Nepal Ministry of Health, Interim Guidance for Reproductive, Maternal, Newborn and Child Health Services in COVID-19 Pandemic | GG | May 21 | <https://nepal.unfpa.org/en/publications/interim-guidance-reproductive-maternal-newborn-and-child-health-services-covid-19> |
| Netherlands | Federation of Medical Specialists and the Dutch Association for Obstetrics and Gynecology, Point of view: COVID-19 and pregnancy, delivery and childbirth | PG | June 17 | <https://www.demedischspecialist.nl/sites/default/files/Standpunt%20COVID-19%20en%20zwangerschap%20en%20bevalling.pdf> |
| New Zealand (1) | Waitemata District Health Board, COVID-19 Maternity Management | GG | April |  |
| New Zealand (2) | Waitemata District Health Board, COVID-19 Cesarean Protocol | GG | April |  |
| Niger | Republic of Niger Ministry of Public Health, Promotion, Protection and Support of Infant and Young Child Feeding in the Context of COVID-19 | GG | April |  |
| Nigeria | Nigerian Ministry of Health and Nigeria Centre for Disease Control, Guidelines for the Management of Pregnant Women and Nursing Mothers | GG | N.D. | <https://covid19.ncdc.gov.ng/media/files/GuidelinesforMgtofPregnantWomen_t5skUIm.pdf> |
| Norway (1) | National Advisory Unit for Breastfeeding (NKA) and the Norwegian Directorate of Health, in collaboration with the National Institute of Public Health (NIPH) and the Norwegian Pediatric Association (NBF), Mothers with Suspected or Proven COVID-19 Infection: Breastfeeding and Infection Preventative Measures | GG/PG | July 1 |  |
| Oman | Sultanate of Oman Ministry of Health, Pregnancy and Coronavirus (COVID-19) Infection Guideline | GG | July | <https://www.moh.gov.om/documents/10194/3903020/COVID+in+pregnancy+16th+July+2020.docx/017453ce-afbd-45e5-087e-21acdd2a3541> |
| Pakistan | Government of Pakistan Ministry of National Health Services, Regulations and Coordination, Guidelines: Sexual, Reproductive and Maternal Health Services during COVID 19 | GG | July 7 | <http://www.nhsrc.gov.pk/SiteImage/Misc/files/20200707%20Interim%20Guidance%20for%20Continuity%20of%20SRH%20Services_2201.pdf> |
| Panama | Panamanian Society of Obstetrics and Gynecology, Guidelines for the Management of those Pregnant with COVID-19 | PG | N.D. | <http://minsa.b-cdn.net/sites/default/files/publicacion-general/manejo_de_pacientes_embarazadas_intrahospitalariamente__0.pdf> |
| Paraguay (1) | Paraguay Ministry of Public Health and Social Welfare, Protocol for COVID-19: Management Guide for Pregnant Women and Obstetric Events | PG | May 7 | <https://www.mspbs.gov.py/dependencias/portal/adjunto/3ee4c9-GUIADEMANEJODEMUJERESEMBARAZADASYEVENTOSOBSTETRICOS.pdf> |
| Paraguay (2) | Paraguay Ministry of Public Health and Social Welfare, Protocol for COVID-19 Neonatal Care | PG | May 28 |  |
| Philippines | Republic of the Philippines Ministry of Health, Interim Guidelines on COVID-19 Management of Pregnant Women, Women about to Give Birth, and Newborns | GG | July 13 | <https://doh.gov.ph/sites/default/files/health-update/dm2020-0319.pdf> |
| Poland | Republic of Poland Ministry of Health, Recommendations on How to Deal with the Current Epidemiological Situation in the Case of Newborns of Mothers Infected or Suspected of COVID-19 | GG | September 29 | <https://www.gov.pl/web/zdrowie/wytyczne-dla-poszczegolnych-zakresow-i-rodzajow-swiadczen> (Number 20) |
| Portugal | Portugal National Health Service, COVID-19: Pregnancy and Childbirth | GG | May 19 | <https://www.dgs.pt/normas-orientacoes-e-informacoes/orientacoes-e-circulares-informativas/orientacao-n-0182020-de-30032020-pdf.aspx> |
| Romania | Government of Romania Ministry of Health, Methodology Regarding Birth in Pregnancies with Infection with SARS-CoV-2/COVID-19 Infection Suspected/Confirmed, Taking Up, Care and Medical Assistance for the New Baby | GG | October 26 | <https://sogr.ro/actualizare-26-10-2020-metodologia-privind-nasterea-la-gravidele-cu-infectie-suspicionata-confirmata-cu-sars-cov-2-covid-19-preluarea-ingrijirea-si-asistenta-medicala-a-nou-nascutului/> |
| Russia | Russian Federation Ministry of Health Methodological Recommendations Organization of Medical Care of Pregnant Women, Women in Labor, Parturient Women and Newborns with New Coronavirus COVID-19 Infections Version 2 | GG | May |  |
| Saudi Arabia (1) | Kingdom of Saudi Arabia Ministry of Health, Guidelines for Neonates Born to Mothers with Suspected or Confirmed COVID-19 Version 3 | GG | September 1 | <https://www.moh.gov.sa/Ministry/MediaCenter/Publications/Documents/Saudi-Guideline-for-Neonate.pdf> |
| Saudi Arabia (2) | Kingdom of Saudi Arabia Ministry of Health, Breastfeeding Promotion During the COVID-19 Pandemic | GG | April 28 | <https://www.moh.gov.sa/Ministry/MediaCenter/Publications/Documents/Interim-Guidance-Final.pdf> |
| Saudi Arabia (3) | Kingdom of Saudi Arabia Ministry of Health, Clinical Guidelines for Nursing and Midwifery Practice during the Coronavirus (COVID-19) Pandemic | GG | N.D. | <https://www.moh.gov.sa/en/Ministry/MediaCenter/Publications/Documents/Clinical-Guidelines-for-Nursing-Midwifery-Practice-during-COVID-19-Pandemic.pdf> |
| Saudi Arabia (4) | Kingdom of Saudi Arabia Ministry of Health, COVID-19 in Pregnancy Rapid Response Guidelines Version 1.1 | GG | N.D. | <https://www.moh.gov.sa/Ministry/MediaCenter/Publications/Documents/COVID-19-IN-PREGNANCY-MOH-Protocol.pdf> |
| Serbia | Republic of Serbia Ministry of Health Expert Commission for Breastfeeding Support, Instructions for Care and Nutrition of New Mothers who have a Suspected or Confirmed COVID-19 Infection in Hospital or Home Isolation | GG | N.D. | <https://www.batut.org.rs/download/aktuelno/Uputstvo%20za%20negu%20i%20ishranu%20novorodjencadi%20COVID%20pozitivnih%20majki.pdf> |
| Singapore | Academy of Medicine Singapore and College of Obstetricians and Gynaecologists Singapore, Management of Pregnancy and Birth in Women with Coronavirus Disease | PG | April | <https://www.ams.edu.sg/view-pdf.aspx?file=media%5c5444_fi_255.pdf&ofile=(Committee+Opinion)+Management+of+Coronavirus+disease+(COVID-19)+in+Pregnancy+April+2020+0420+final.pdf> |
| Slovakia (1) | Zibolen and Matasova, Newborn Care of Mothers with Suspected or Confirmed COVID-19 Infection | GG | November 24 | <https://www.health.gov.sk/Clanok?Hlavna-sprava-COVID-19> |
| Slovakia (2) | Borovsky and Kristufkova, General Recommendations for Delivery Rooms | GG | N.D. | <https://www.health.gov.sk/Clanok?Hlavna-sprava-COVID-19> |
| Slovakia (3) | Borovsky and Kristufkova, Delivery of a Mother with Suspected/Confirmed COVID-19 Infection | GG | N.D. | <https://www.health.gov.sk/Clanok?Hlavna-sprava-COVID-19> |
| Slovenia | Department of Perinatology University Medical Center, Recommendations for Professional Treatment of Pregnant Women and Newborn Children During the COVID-19 Epidemic | PG | October 27 | <https://www.nijz.si/sites/www.nijz.si/files/uploaded/covid7-porodnisnice-za_strokovno_javnost_verzija7.pdf> |
| South Africa | South African Framework and Guidelines for Maternal and Neonatal Care during a Crisis  COVID-19 response | GG | July 8 | <https://www.samrc.ac.za/sites/default/files/attachments/2020-08-20/COVID19Maternalnewborncareguidelines.pdf> |
| South Korea | Korean Society of Pediatric Infectious Diseases, Coronavirus Infectious Disease 19 Response Guidelines (Newborn, Infants, Children, and Adolescents) | PG | March 20 | <https://web.archive.org/web/20201129003645/http:/www.kspid.or.kr/board/list.html?code=notice&num=553> |
| South Sudan | South Sudan Ministry of Health, COVID-19 Clinical Care Management Guideline for South Sudan | GG | July 2 | <https://moh.gov.ss/case_mgt.php> |
| Spain | Government of Spain Ministry of Health, Management of the Pregnant Woman and the Newborn with COVID-19 | GG | June 17 | <https://www.mscbs.gob.es/profesionales/saludPublica/ccayes/alertasActual/nCov/documentos/Documento_manejo_embarazo_recien_nacido.pdf> |
| Sweden | Swedish Society of Obstetrics and Gynecology and Swedish Neonatal Society, Recommendation on Handling of Pregnant and Newborn Children to Women with Verified / Probable Covid-19 | PG | April 5 | <https://www.sfog.se/media/336929/sfog-raad-om-handlaeggning-av-gravida-och-barn-till-kvinnor-med-verifieradelsannolik-covid-19_ver-2_200405.pdf> |
| Switzerland | Swissnoso in Cooperation with Pediatric Infectious Disease Group of Switzerland, Interim Preventative Measures in Hospitals for a Hospitalized Patient with Justified Suspicion of with a Confirmed COVID-19 Infection | PG | October 29 | <https://www.swissnoso.ch/fileadmin/swissnoso/Dokumente/5_Forschung_und_Entwicklung/6_Aktuelle_Erreignisse/201029_Vorsorgemassnahmen_COVID-19_Spital_Paediatrie_V5.2.pdf> |
| Taiwan | Taiwan Centers for Disease Control, Interim Guidelines for Clinical Management of Novel Coronavirus (SARS-CoV-2) Infection | GG | August 17 | <https://www.cdc.gov.tw/File/Get/ilsn3l4FfxbmtxLCMwFidw> ^c^ |
| Thailand | Royal Thailand College of Obstetrics and Gynecology Clinical Practice Guideline Management of Covid-19 Infection in Pregnancy 3 July 2020 | PG | July 17 | <http://www.rtcog.or.th/home/wp-content/uploads/2020/07/CPG-Covid-Preg-V3-July20.pdf> |
| Timor Leste (1) | Democratic Republic of Timor Leste Ministry of Health, Guidelines for Antenatal Care for Women with COVID-19 in Timor Leste | GG | April 1 |  |
| Timor Leste (2) | Democratic Republic of Timor Leste Ministry of Health, Guidelines for Intrapartum and Immediate Post-Partum Care for Women with COVID-19 in Timor Leste | GG | April 1 |  |
| Turkey (1) | Turkish Republic Ministry of Health, COVID-19 (SARS-CoV-2 Infection) Pregnancy Follow Up in Health Institutions in the Period when Respiratory System Diseases are Widespread | GG | October 16 |  |
| Turkey (2) | Turkish Republic Ministry of Health, COVID-19 (SARS-CoV-2 Infection) Child Patient Management and Treatment | GG | September 1 |  |
| Uganda | Republic of Uganda Ministry of Health, National Guidelines for Management of COVID-19 | GG | April 23 | <http://library.health.go.ug/publications/disease-survielance-outbreaks/national-guidelines-managing-covid-19-april-23-2020> |
| United Kingdom | Royal College of Obstetrics and Gynaecology and Royal College of Midwives, Coronavirus (COVID-19) in Pregnancy V. 12 | PG | October 14 |  |
| Ukraine | Ukraine Ministry of Health Standards of Medical Care Coronavirus Disease (COVID-19) | GG | March 28 | <https://moz.gov.ua/uploads/5/26472-standarti_medicnoi_dopomogi_pri_kovid.pdf> |
| United States of America (1) | US Centers for Disease Control and Prevention, Considerations for Inpatient Obstetric Healthcare Settings | GG | May 20 | <https://www.cdc.gov/coronavirus/2019-ncov/hcp/inpatient-obstetric-healthcare-guidance.html> |
| United States of America (2) | US Centers for Disease Control and Prevention, Evaluation and Management Considerations for Neonates at Risk for COVID-19 | GG | August 3 | <https://www.cdc.gov/coronavirus/2019-ncov/hcp/caring-for-newborns.html> |
| Uruguay | Republic of Uruguay Ministry of Public Health, Recommendations Related to Assistance of the Pregnant Woman and the Newborn in the COVID-19 Pandemic | GG | April 23 | <https://www.gub.uy/ministerio-salud-publica/comunicacion/publicaciones/recomendaciones-para-mujer-embarazada-recien-nacido-frente-covid-19> |
| Venezuela (1) | Republic of Venezuela Ministry of the Popular Power for Health, COVID-19 Recommendations for the Comprehensive Care of Women, Pregnant Women and Newborns in the Boliavarian Republic of Venezuela | GG | April 24 | <http://www.mpps.gob.ve/index.php/sistemas/cheques> |
| Venezuela (2) | Republic of Venezuela Ministry of the Popular Power for Health, COVID-19, pregnancy and breastfeeding | GG | March 13 | <http://www.mpps.gob.ve/index.php/sistemas/cheques> |
| Vietnam | Socialist Republic of Vietnam Ministry of Health, Decision Promulgating the Guidance on Temporary Promotion for, and Handling of, Acute Inhabitic Infection with SARS-CoV-2 (COVID-19) Infection in Pregnant Women and Newborn Children | GG | March 21 |  |
| Zimbabwe | Zimbabwe Society of Obstetricians and Gynaecologists, COVID-19 and Pregnancy | PG | March |  |
| **International Guidance** | **Title of document (translated)** | **Type ^a^** | **Date released (2020)** | **Source ^b^** |
| PJIMT | Preparing for COVID-19 in the PICs: Guidance for essential ANC, intrapartum, and PNC services | IO | July 6 |  |
| PAHO | Breastfeeding and Covid-19 | IO | May 20 | <https://iris.paho.org/bitstream/handle/10665.2/52298/PAHONMHRFCOVID-19200018_eng.pdf?sequence=1&isAllowed=y> |

*Note*: ^a^ Type = type of document; GG = government guidance document; PG = professional medical association guidance document; IO = International organization; N.D .= no date known; PJMIT= Pacific Joint Incident Management Team; PAHO= Pan American Health Organization; ^b^ empty cells are documents we obtained from the sources that are not on-line; can be requested if needed;  ^c^ found on Internet Archive Wayback Machine (https://archive.org/); all websites are accurate as of August 17, 2021.
